# Nutrient assessment of sea buckthorn residues as potential feed ingredients

**DOI:** 10.3389/fvets.2026.1767594

**Published:** 2026-02-27

**Authors:** Dunja Malenica, Rajeev Bhat, Marko Kass, Ragnar Leming, Larissa Silva Maciel, Koit Herodes, Meelis Ots

**Affiliations:** 1 Institute of Veterinary Medicine and Animal Sciences, Estonian University of Life Sciences, Tartu, Estonia; 2 Centre of Estonian Rural Research and Knowledge, Jõgeva, Estonia; 3 Institute of Chemistry, University of Tartu, Tartu, Estonia

**Keywords:** agro-industrial by-products, animal nutrition requirements, circle feed, feed ingredient evaluation, metabolizable energy prediction, waste management

## Abstract

This study aimed to evaluate the suitability of sea buckthorn by-products, including pomace without seeds (SBPW), pomace with seeds (SBPS), and leaves (SBL) as potential livestock feed ingredients. Proximate composition, amino acids, fatty acids, minerals, vitamins, and metabolizable energy (ME) were assessed and compared to the daily requirements of livestock, assuming an inclusion rate of 2.5% of dry matter intake in different animal species. SBPW exhibited the highest metabolizable energy (13.7–17 MJ/kg DM across species), exceeding 2.5% of daily requirements in most animal species, suggesting it is a good energy source, whereas SBL contributed the least (8.1–13.6 MJ/kg DM across species). SBL was mineral rich, with notably elevated Fe, Ca, Mg, and Mn concentrations, supporting its use as a mineral supplement, whereas both pomaces contained low mineral levels. SBL and especially SBPS revealed promising amino acid profiles, showing potential as protein source, meeting 2.5% daily amino acid requirements across most categories, especially gilts and pregnant sows. SBPS contained high levels of arginine (31.4 g/kg DM) and lysine (11.3 g/kg DM), while SBL also supplied substantial amounts of essential amino acids (e.g., lysine 10.2 g/kg DM). Sulfur-containing amino acids were limiting in all residues, with methionine especially low (e.g., SBPW 0.9 g/kg DM). SBPW provided insufficient essential amino acids for most categories, suggesting it would have to be combined with protein feeds to achieve a balanced ration. SBPS was rich in polyunsaturated (notably alpha-linolenic: 1.39 g/kg DM) fatty acids, while SBPW contained more monounsaturated and SBL more saturated fats. Both pomaces provided similar linoleic acid (≈2.5 g/kg DM), exceeding pig but not poultry requirements at 2.5% dry matter intake. All residues were rich in vitamin E, particularly SBPW (720 mg/kg DM), and pomaces contained substantial β-carotene (SBPW 258 mg/kg DM, SBPS 151 mg/kg DM), with potential benefits for gestating animals and those experiencing high oxidative stress. Overall, this study provides an initial assessment of sea buckthorn residues as alternative feed resources and offers guidance for their strategic inclusion in animal diets supporting more sustainable, circular feeding systems.

## 1 Introduction

Feed industries have faced a number of challenges in recent years: deficit of land availability, water accessibility, soil degradation, human population growth and urbanization, food-fuel-feed competition and rising costs and shortages of conventional feed ingredients (1, 2). The impact of climate change exacerbates these issues even further, impacting both the availability and quality of feed resources worldwide (3). Globally, livestock production uses up to 40% of arable land for feed crop production, and over 30% of cereal production is allocated to animal feeds, contributing significantly to environmental pressures including greenhouse gas emissions, biodiversity loss, and water and resource usage (4, 5). These challenges highlight the urgent need for sustainable, cost-effective, and environmentally responsible alternatives to conventional livestock feeds, making this an issue of global significance for animal agriculture and food security.

One potential solution to these problems is the use of agro-industrial by-products as alternative feed ingredients. Agro-industrial by-products are a rich source of a range of nutrients and bioactive compounds and thus represent a low-cost feed option, which could promote animal health and productivity (6). In addition, their use in animal diets could reduce environmental pollution by offering an alternative to current disposal methods which contribute to greenhouse gas emissions (6–8). Furthermore, increasing circularity in EU food systems is a strategic priority, and nutrient recovery through animal nutrition is central to the concept of circular feed (9).

Among agro-industrial by-products, sea buckthorn (SB) (*Hippophae rhamnoides* L.) residues show particular promise. This hardy, rapid-growing shrub of the Elaeagnaceae family grows widely in North America, Europe, and Asia (10–12). Its berries and leaves are rich in secondary metabolites with known antioxidant, cardioprotective, antimicrobial, anti-inflammatory, and hepatoprotective properties (13, 14). While SB berries are primarily processed for juice and oil production, large quantities of residues—including pomace with seeds (SBPS), pomace without seeds (SBPW), and leaves (SBL)—are typically discarded (15). Given their nutrient content and bioactive compounds, these residues warrant investigation as potential livestock feed ingredients.

Additionally, although a few feeding trials have used SB pomace or leaves in livestock diets, the data remain fragmented, and there is still a clear gap in the literature regarding the evaluation of SB by-products’ nutrient profiles in regard to animal feeding, and comparisons with various animal nutrient requirements (16–18). A comprehensive evaluation of SB residues is therefore needed to support evidence-based diet formulation and to identify potential nutritional imbalances that may arise from their long-term or large-scale use in livestock feeding systems. The present study provides an initial step toward this goal. By integrating multi-nutrient profiling with livestock nutrient requirements, this study provides a novel, comprehensive first-step screening of SB by-products as alternative feed resources. It explores patterns in how these nutrient profiles align with the nutritional requirements of selected livestock species (poultry, pigs, horses, and cows) and their respective production categories (e.g., growing, lactating, gestating, working animals), identifying nutrient strengths, deficiencies, and potential suitability among different species. In addition, it provides a foundation for future experimental feeding and digestibility trials.

This study is looking for a solution which could help reduce animal feed costs and reliance on synthetic supplements while at the same time minimizing the environmental impacts of unsustainable disposal of these by-products. In the long term, such efforts could support more balanced, sustainable feeding strategies across production systems.

## 2 Materials and methods

### 2.1 Sample preparation

All SB materials were obtained from Trocos Trade OÜ (Tartu, Estonia). Oven-drying was performed at 60 °C using a Binder ED 30 gravity convection drying chamber (Tuttlingen, Germany). For amino acid analyses, samples were freeze-dried using a Martin Christ Alpha 3–4 LSC basic freeze dryer (Osterode am Harz, Germany).

### 2.2 Proximate analyses

For proximate analyses standard methods of the Association of Official Analytical Chemists were followed (19). To measure dry matter (DM) content, samples were heated for 2 h in an oven at 130 °C to constant weight. For determining crude ash, samples were burned at 550 °C for 18 h. Petroleum ether extraction was used to determine crude fat (EE), employing a Soxtec System 2043 Extraction Unit (FOSS, Hillerød, Denmark). The Kjeldahl method, performed with a Kjeltec 2300 Analyzer (FOSS, Hillerød, Denmark), was used to determine crude protein (CP) based on the nitrogen (N) content using a conversion factor of 6.25 (CP = N × 6.25). Crude fiber (CF) analysis was conducted in accordance with ISO 6865:2000 (20). Neutral Detergent Fiber (NDF) and Acid Detergent Fiber (ADF) were evaluated with a fiber analyzer ANKOM220 (ANKOM Technology, Macedon NY, USA) following a method described by Van Soest et al. (21). For determining starch content of samples, the method adopted by the AOAC (Official Method 996.11) and AACC (Method 76.13.01) was used (22, 23). The nitrogen-free extractives (NFE) fraction was derived by estimation of difference, using the Equation 1:


NFE(%)=DM−(crudeash+CP+CF+EE). (1)

### 2.3 Mineral content

An Agilent 5800 ICP-OES was used to determine the contents of 10 macro- and microelements: calcium (Ca), phosphorus (P), potassium (K), magnesium (Mg), sodium (Na), iron (Fe), copper (Cu), zinc (Zn), manganese (Mn) molybdenum (Mo) and cobalt (Co). The procedure followed the Agilent Application Note for Food and Agriculture (Method 5,110 VDV ICP-OES) (24). In brief, to prepare the samples, approximately 0.5 g of each sample was weighed into a PTFE test tube followed by adding 2.5 mL of concentrated HNO_3_ and 2.5 mL of concentrated HCl. The samples were then subjected to microwave digestion with closed cup using Mars 6 microwave (CEM Corporation, Matthews, USA). Following digestion, the samples were diluted to a final volume of 50 mL with distilled water. Working standards were prepared from single element Agilent ICP-OES Calibration Standards. IAG Ringtest samples (tested by 28 feedingstuff laboratories) were used as control samples. All elements were quantified using linear calibration curves, which demonstrated high correlation coefficients (lowest *R*^2^ = 0.999), as it can be seen in Supplementary Table S1.

### 2.4 Amino acids

Amino acid concentrations were determined using liquid chromatography–tandem mass spectrometry, following an adapted methodology previously reported by Ben-Othamn et al. (25). Amino acids were quantified after acid hydrolysis of the samples, ensuring that the results represent amino acids released from hydrolyzed proteins rather than just free amino acids. Prior to chromatographic determination, amino acids were derivatized to enable accurate detection and separation by RPLC-ESI-MS/MS. The LC MS/MS system consisted of an Agilent 1290 Infinity II quaternary pump, a column thermostat, an autosampler, and an Agilent 6460 Triple Quadripole mass spectrometer, equipped with an Agilent Jet stream Technology electrospray ionization source. A Zorbax Eclipse Plus C18 column (3.0 × 100 mm, 1.8 μm) was used for the chromatographic separation coupled with a guard column (3.0 × 5 mm, 1.8 μm). The mobile phase consisted of 0.1% formic acid in water (solvent A) and acetonitrile (solvent B), delivered at a flow rate of 0.4 mL/min. The gradient program was as follows: 0–2 min, 10% B; 2–27 min, 10–98% B; 27–29 min, 98% B; and 29–31 min, 98–10% B. Positive ionization mode with a capillary voltage of 3,000 V in dynamic multiple reaction monitoring mode was used for analysis (25). Amino acid concentrations were converted to g per kg of dry matter (DM) by dividing the obtained concentration values in the solution by the DM content of the freeze-dried samples, enabling direct comparison with the animals’ requirements.

### 2.5 Fatty acids

Fatty acid contents of SB residues were determined following the methodology previously described by Sukhija et al. (26). Oven dried samples (50–500 mg of sample depending on fat content, ca. 10–60 mg of fat) were placed in 16 mL test tubes, and 0.75 mL of internal standard heptadecanoic acid (C17:0) solution in toluene (4 mg/mL), 0.75 mL toluene, and 2.25 mL freshly prepared 5% HCl in methanol were added. The mixture was vortexed for 1 min, heated at 70 °C for 2 h, and cooled to room temperature. Neutralization was achieved with the addition of 3.75 mL 6% aqueous K₂CO₃ and 1.5 mL toluene, followed by mixing and centrifugation (5 min, 1,500×*g*). The upper layer was transferred to a new tube, treated with ~0.75 g anhydrous Na₂SO₄ and approximately 0.75 g activated carbon, mixed briefly, and left to stand for 1 h. After centrifugation (5 min, 4,000×*g*), a clear toluene layer containing 10–30 mg/mL fatty acid methyl esters (FAMEs) was obtained. Complete methylation was verified using thin-layer chromatography (TLC) with hexane:diethyl ether:acetic acid (85:15:1) as the eluent, visualized under iodine vapor or UV light.

Following this, FAMEs were analyzed on an Agilent 6890 GC (Santa Clara, CA, USA), equipped with a split/splitless injector and flame ionization detector, using a CP-Sil 88 capillary column (100 m × 0.25 mm i.d., 0.20 μm film). Hydrogen was the carrier gas. The injector and detector were set at 260 °C and detector gases were supplied at 30 mL/min H₂ and 300 mL/min air. A 1 μL sample (15–40 mg/mL) was injected with a split ratio of 60:1. The oven program was 170 °C for 10 min, ramped at 4 °C/min to 240 °C, held for 10 min, with constant carrier gas pressure of 25 psi. Fatty acids were identified by comparing retention times with commercial FAME standards (Supelco 37 Component Mix, Nu-Chek Prep standards, FAME Mix, and individual FAMEs). Conjugated linoleic acid isomers were identified using Nu-Chek Prep standards. Measured values of short-chain fatty acids (C4:0–C12:0) were corrected using the correlation factors provided in the Supplementary Material (Supplementary Table S2).

### 2.6 Vitamin content

The determination of vitamins A, D, E, B_1_, B_2_, B_3_ and B_6_ was performed using Agilent HPLC 1200, fitted with quaternary pump, autosampler, column thermostat, and diode-array (DAD) and fluorescence detectors (FLD). Fat-soluble vitamins were detected with the mobile phase consisting of water, methanol and acetonitrile, while for water-soluble vitamins it consisted of acidic water and methanol. The flow rate was 0.5 mL/min. Vitamins B_1_, B_2_, and B_3_ were quantified using DAD, while FLD was used for vitamin B_6_. The assessment of B_5_ vitamin content was carried out with LC–MS/MS, which was operated in positive ionization mode with capillary voltage of 3,500 V. Analyses were conducted according to accredited European standard methods with some modifications: EVS-EN 12823–1:2014 for vitamin A (27), EVS-EN 14122:2014 for vitamin B1 (28), EVS-EN 14152:2014 for vitamin B2 (29), EVS-EN 15652:2009 for B3 (30), EVS-EN 14663:2006 for vitamin B6 (31), EVS-EN 12821:2009 for vitamin D (32), and EVS-EN 12822:2014 for vitamin E (33). The concentrations were determined by calculating the concentration from the peak area observed in the sample, using external calibration.

Values below the detection limits (LOD), which were set by the analytical method, are reported as “<.” For vitamin A, the LOD was 0.02 μg/g for most samples, whereas SBPS, which was analyzed at a later stage, had an LOD of 0.05 μg/g. For vitamin D, the LOD was 0.01 μg/g for most samples and 0.005 μg/g for SBPS. The LOD for vitamin B_1_ was 0.1 μg/g for all samples.

### 2.7 Conversion of β-carotene to vitamin A

Species-specific protocols were applied to estimate the potential conversion of β-carotene to vitamin A. For pigs, the National research council (NRC) (2012) indicates that 1 mg of β-carotene has a calculated potency of 0.08 mg vitamin A; therefore, β-carotene values of the samples were multiplied by 0.08 (34). For ruminants, in accordance with the NorFor system, a conversion factor of 1 mg of β-carotene = 400 IU (0.12 mg) of vitamin A was applied (35). Based on NRC (2007), the same factor was used for horses (36). For poultry, the conversion efficiency is higher, with a 3:1 ratio, corresponding to 1 mg of β-carotene being equivalent to 0.33 mg of vitamin A (37).

### 2.8 Acid-Detergent Insoluble Protein

For determination of Acid-Detergent Insoluble Protein (ADIP), ADF was first measured according to the AOAC method 973.18 (38). The CP content of the residue remaining after ADF determination was then analyzed using the Kjeldahl method. ADIP in the DM of the sample were calculated using the Equation 2:


$$\text{ADIP},\%\mathrm{DM}=\frac{\text{ADFCP}(\mathrm{DM})\times \mathrm{ADF}\;(\mathrm{DM})}{100}$$ (2)

Where ADFCP represents the crude protein content of the remaining residue after ADF has been performed.

To express ADIP as a percentage of the sample’s CP, the Equation 3 was used:


$$\text{ADIP},\%\mathrm{CP}=\frac{\%\text{ADIP}\;(\mathrm{DM})\times 100}{\%\mathrm{CP}\;(\mathrm{DM})}$$ (3)

### 2.9 Gross energy and metabolizable energy predictions

Gross Energy (GE) of the by-products was estimated using calorific coefficients of different nutrients as described by Equation 4 (39):


GE=(23.9×CP+39.8×EE+20.1×CF+17.5×NFE)/100 (4)

Obtained values for GE of samples were expressed as MJ/kg DM. Due to the absence of direct measurements on the digestibility and energy availability of the SB by-products, metabolizable energy (ME) values for different animal species were estimated using prediction equations based on the chemical composition of the samples (Equations 5–8). For ruminants, ME was estimated using both the chemical composition and total gas production (24 h) obtained from *in vitro* gas production evaluation. Although the gas production data are not shown, they were included in the calculations.

For pigs, the following prediction Equation 5 was applied (40):


ME=1133+0.65×GE−29.05×ash−23.17×NDF (5)

In this equation, NDF and ash contents were expressed as % DM. GE was estimated using Equation 3 and while it was originally expressed in MJ/kg DM; these values were converted to kcal/kg DM to be compatible with the ME equations. Final ME values expressed as kcal/kg DM were converted to MJ/kg DM, through multiplication with a factor of 0.004187.

For broilers, the apparent metabolizable energy corrected for nitrogen (AMEn) was estimated using the following Equation 6 developed by Alvarenga et al. (41):


AMEn=4095.41+56.84×EE−225.26×ash−22.24×NDF (6)

Equation 6 was used for energy and protein concentrate feedstuffs with nutrient inputs expressed as % of DM. The obtained AMEn values of by-products were expressed in kcal/kg DM and subsequently converted to MJ/kg DM using a factor of 0.004187.

For horses, metabolizable energy was estimated using the Equation 7 proposed by Kienzle et al. (42):


$$\begin{array}{l} \mathrm{ME}=-3.54+0.0129\times \mathrm{CP}+0.0420\times \mathrm{EE}-0.0019\times \mathrm{CF}\\ +0.0185\times \mathrm{NFE} \end{array}$$ (7)

In Equation 7, all crude nutrients were expressed in g/kg of DM while obtained ME results were expressed as MJ/kg DM.

Metabolizable energy in ruminants was estimated using the Equation 8 developed by Menke et al. (43):


$$\begin{array}{l} \mathrm{ME}=1.06+0.1570\times \mathrm{Gas}\;\text{produced}+0.0084\times \mathrm{CP}+0.022\times \mathrm{EE}\\ –0.0081\times \mathrm{Ash}. \end{array}$$ (8)

Total gas production was expressed as mL/200 mg DM, while nutrients included in Equation 8 were expressed as g/kg DM. Final ME results were expressed as MJ/kg DM.

### 2.10 Comparison of SB residue nutritional profile with conventional feed ingredients

The proximate composition, fatty acid, amino acid, mineral and vitamin contents of the samples were compared to reference values of conventional feed ingredients from INRA and NorFor databases, with the aim of identifying relative differences (i.e., whether values were higher or lower) (44, 45). The following conventional feed ingredients were used for comparison: cereals, legumes, oilseed and oilseed by-products and roughages. Detailed information regarding the selected conventional feed ingredients and their chemical composition is provided in Supplementary Tables S7–12. Since ADIP values were not provided in either the NorFor or INRA feed tables, the ADIP content of the materials was compared with that of conventional feed ingredients, using data from McDonald et al. (37).

### 2.11 Species-specific nutrient requirements

To compare SB residues nutritional profiles against animals’ nutritional requirements, multiple feed evaluation systems were used, with the systems near the Baltic regions prioritized: Natural Resources Institute Finland (Luke) for poultry, horses and ruminants (46) and the Danish nutrient standards for pigs (47). Nutrients which were not covered in these systems were derived from NRC (34, 36, 48) and the Estonian system for horses, pigs and cattle (49, 50). Daily nutrient requirements were applied directly, while values reported per 1 kg of feed/DM were converted to daily amounts using estimated feed or DM intake.

To assess how well the nutrients in the samples meet the daily requirements of various animal species and categories, SB residues were assumed to be included at a uniform level of 2.5% of an animal’s daily DMI. This rate was selected considering the nutritional requirements of the target species, the chemical composition of the by-products, and their practical availability. Due to the relatively high fiber content of SB materials, their inclusion in the diets of poultry and weaned piglets is limited, as these species are unable to digest fiber efficiently. Moreover, while larger animals could theoretically tolerate higher inclusion rates of SB residues, this would mean that daily intakes of SB residues would exceed 1 kg per animal, which could be impractical and unsustainable. This is because the availability of SB residues is highly seasonal, depending on the processing period of berries, and large quantities may not be available throughout the year. In addition, the residues are high in moisture, requiring drying or other preservation methods to ensure stability and prevent microbial spoilage in case of large quantities. While small quantities could hypothetically be dried/preserved more easily using simple on-farm drying or fed fresh, managing large amounts would require dedicated additional infrastructure for drying and preservation which many farms may not be able to provide. This makes large-scale utilization less practical and potentially less sustainable. Therefore, 2.5% level was applied across all species to ensure both practicality and comparability.

Percentage fulfillment of daily nutrient requirements was calculated by estimating specific nutrient intake at a 2.5% inclusion rate of each SB residue in the animals’ diets. The resulting nutrient supply (g or mg/day) was then divided by the corresponding daily requirement of that nutrient and multiplied by 100, as shown in Equation 9. Contributions <2.5% were considered deficient, ≈2.5% adequate, and values well above 2.5% rich or, if substantially higher, excessive.


$$\begin{array}{l} \text{Percentage fulfilment}=\\ \frac{\text{Nutrient intake from sample}\;\mathrm{at}\;2.5\%\mathrm{DMI}}{\text{Daily requirement of nutrient}}\times 100 \end{array}$$ (9)

For vitamin concentrations below the detection limit (e.g., <20 mg/kg), values were replaced with half of the detection limit (LOD/2; in this case 10 mg/kg) to allow comparison with animal daily nutritional requirements. All vitamin requirements presented as IU/day were converted to mg/day to accomplish an easier comparison with the SB samples’ vitamin contents (51).

Additionally, the assessment of daily amino acid requirement fulfillment was limited to pigs and poultry, as relevant requirement data were only available for these species. For horses, only lysine fulfillment was evaluated, since it was the sole amino acid with established requirement data (36, 46, 52, 53). For ruminants (except calves), amino acid requirements are not specifically defined; therefore, daily requirement fulfillment was not determined. Moreover, such an assessment would be unreliable, as it is not possible to estimate what proportion of amino acids would escape rumen degradation. Regarding fatty acids, only the requirements for linoleic acid were specified for different categories of chickens and pigs, and this was compared with the linoleic acid content of the samples (34, 46).

#### 2.11.1 Nutrient requirements for horses

To assess the samples’ fulfillment of daily nutrient requirements for horses, values published by Luke were used (46). These requirements were based on three horse categories: working horses (ranging from light to heavy exercise), pregnant mares, and lactating mares. For certain nutrients (CP, lysine, Na, K, and Mn) which were not included in Luke, the requirements from NRC were used, as the compatibility between Luke and NRC made this choice appropriate (36, 46). The nutrient requirements, presented as a range in the feed evaluation systems, were averaged to allow a more effective comparison with the nutrient composition of SB samples. The supplementary material presents the detailed nutritional requirements of horses used (Supplementary Table S3).

#### 2.11.2 Nutrient requirements for poultry

To evaluate the samples’ nutrient content against poultry daily requirements, data for broiler chickens (day 23 until slaughter) and laying hens (29–45 weeks of age) were obtained from Luke (46). In the Luke tables, all values were expressed as g/kg or mg/kg. To convert these into daily requirements, feed intake was estimated according to Estonian recommendations for laying hens and 4-week-old broiler chickens (54). For amino acids not reported in Luke, requirements were obtained from McDonald et al. (37). To enable a more effective comparison, the nutrient requirements, which were presented as a range, were averaged. Detailed nutritional requirements of poultry (laying hens and broiler chicken) can be seen in Supplementary Table S4.

#### 2.11.3 Nutrient requirements for pigs

For determining mineral and vitamin requirements of different pig categories, Danish nutrient standards were applied (47). The nutrient content of the samples was compared against the requirements of the following pig categories: weaned piglets (9–15 kg) with standard diet and 1.65–1.8 Danish feed unit for growing pigs (fugp)/kg gain; finishers (30–60 kg) with 2.45–2.6 fugp/kg gain; finishers (75–115 kg) with 2.45–2.6 fugp/kg gain; gilts (60–110 kg and 120 + kg); gestating sows (0–110 days and 110–117 days in gestation) and lactating sows.

Requirements in the Danish nutrient standards were originally expressed per Danish feed unit (FU). For comparison with sample nutrient content, these values were converted to g, mg, or IU/kg according to the instructions provided for each pig category in the Danish nutrient standards. Daily requirements were then obtained by multiplying the obtained values by feed intake values proposed by the NRC (34).

For each pig category, Ca requirements were based on diets supplemented with 150–200% phytase (approximately 390–1,312.5 FTU/kg, depending on the phytase product). Zinc requirements were taken from diets with 0–150% phytase supplementation, as values for 150–200% phytase were not available and this range corresponded most closely to the Ca reference.

Fulfillment of amino acid requirements was evaluated using total daily requirements from the NRC (34), as the Danish Nutrient Standards (47) report only digestible values, and the digestibility of the SB samples’ amino acids is unknown. Linoleic acid requirements were also taken from the NRC (34). To assess protein adequacy, daily CP requirements from the Estonian feeding recommendations were applied, as these were the only values available for total rather than digestible protein (49). The supplementary material shows detailed nutritional requirements for all pig categories (Supplementary Table S5).

#### 2.11.4 Requirements of dairy cows, heifers, dry cows and calves

Nutrient content of the SB samples was evaluated against the daily requirements of a dairy cow weighing 650 kg and producing 35 kg of energy-corrected milk (ECM), heifers weighing 200 kg and 400 kg, dry cows, and 2-month-old calves. Nutritional requirements for dairy cows and heifers were primarily based on Luke recommendations (46). CP requirements were based on NRC values for heifers and Penn State University guidelines for dairy cows (55). Additionally, as K requirements for heifers (200 kg and 400 kg) were not available in the Luke database, they were estimated using the NorFor model’s proposed equation (Equation 10) for the minimum dietary K requirement in growing cattle (35):


Kreq.min(g/day)=6×DMI (10)

To estimate the daily nutrient requirements of heifers and dairy cows, the values provided per kg of DM were multiplied by assumed DMI. For lactating dairy cows, DMI was assumed to be 3.8% of body weight, according to Estonian feeding recommendations (49). For heifers (200 kg and 400 kg), DMI was calculated using the NorFor predictive equation (Equation 11) (35):


$$\begin{array}{l} \mathrm{DMI}\;\text{heifer}=\frac{\mathrm{BW}}{100}\times \\ \left(0.000004\times BW^{2}-0.0049\times \mathrm{BW}+3.1033\right) \end{array}$$ (11)

NRC guidelines were used to compare the nutrient contents of samples against the requirements of dry cows and calves (48). For calves, DMI, methionine, and lysine requirements were based on Estonian feeding recommendations published in the project report *Uuendnoorkarja söötmisstrateegia loomine lüpsikarja efektiivseks ja jätkusuutlikuks taastootmiseks* (50). Detailed nutritional requirements for all categories are presented in Supplementary Table S6.

### 2.12 Statistical analysis

The fulfillment of daily nutrient requirements (%) across different animal categories was calculated using the Microsoft Excel 2017 Data Analysis Add-in. All graphs were generated using R software (v4.1.2; R Core Team, 2021). Tukey’s tests and correlation analyses were performed using R statistical software (v4.1.2; R Core Team, 2021).

To calculate the standard deviation when two amino acids were summed (e.g., methionine + cysteine), the following formula (Equation 12) was used:


$$\text{stdev}\;\mathrm{met}+\mathrm{cys}=\sqrt{\text{stdev}\;\mathrm{me}t^{2}+\text{stdev}\;\mathrm{cy}s^{2}}$$ (12)

## 3 Results

### 3.1 Proximate analyses

**Figure 1:**
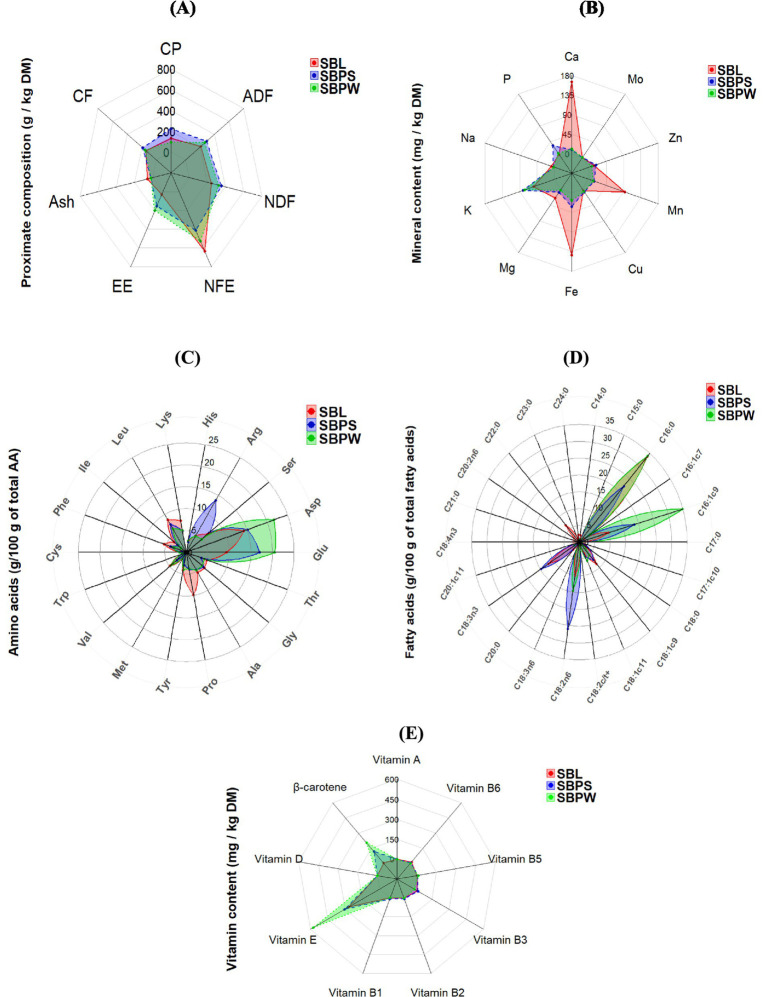
Comparative radar chart of proximate analyses **(A)**, minerals **(B)**, amino acids **(C)**, fatty acids **(D)**, and vitamins **(E)** in sea buckthorn pomace with seeds (SBPS) and sea buckthorn pomace without seeds (SBPW).

**Figure 2:**
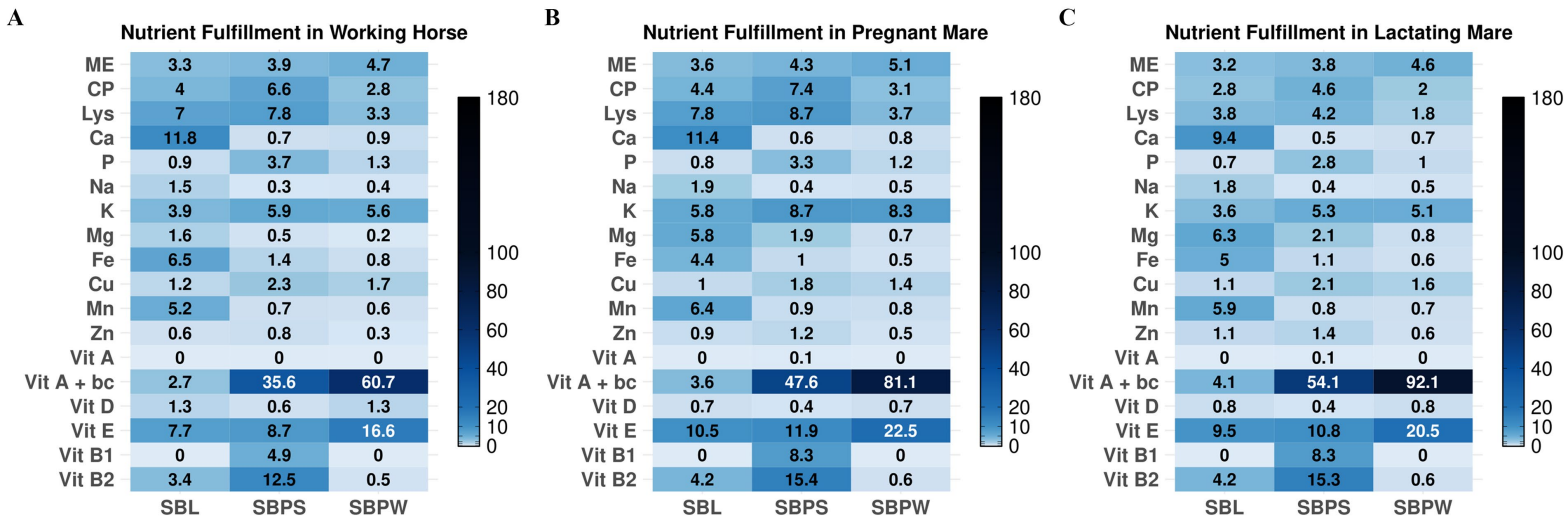
Percentage fulfillment of daily nutrient requirements in different categories of horses: working horse **(A)**, pregnant mare **(B)**, and lactating mare **(C)**, with 2.5% inclusion of sea buckthorn leaves (SBL), sea buckthorn pomace with seeds (SBPS), and sea buckthorn pomace without seeds (SBPW) in DMI. Interpretation: <2.5%: deficient; ≈2.5%adequate; >2.5% rich or, if substantially higher, excessive. ME = metabolizable energy. CP = crude protein, Vit A + bc = vitamin A + hypothetical conversion of carotene to Vitamin A if we assume full conversion.

**Figure 3:**
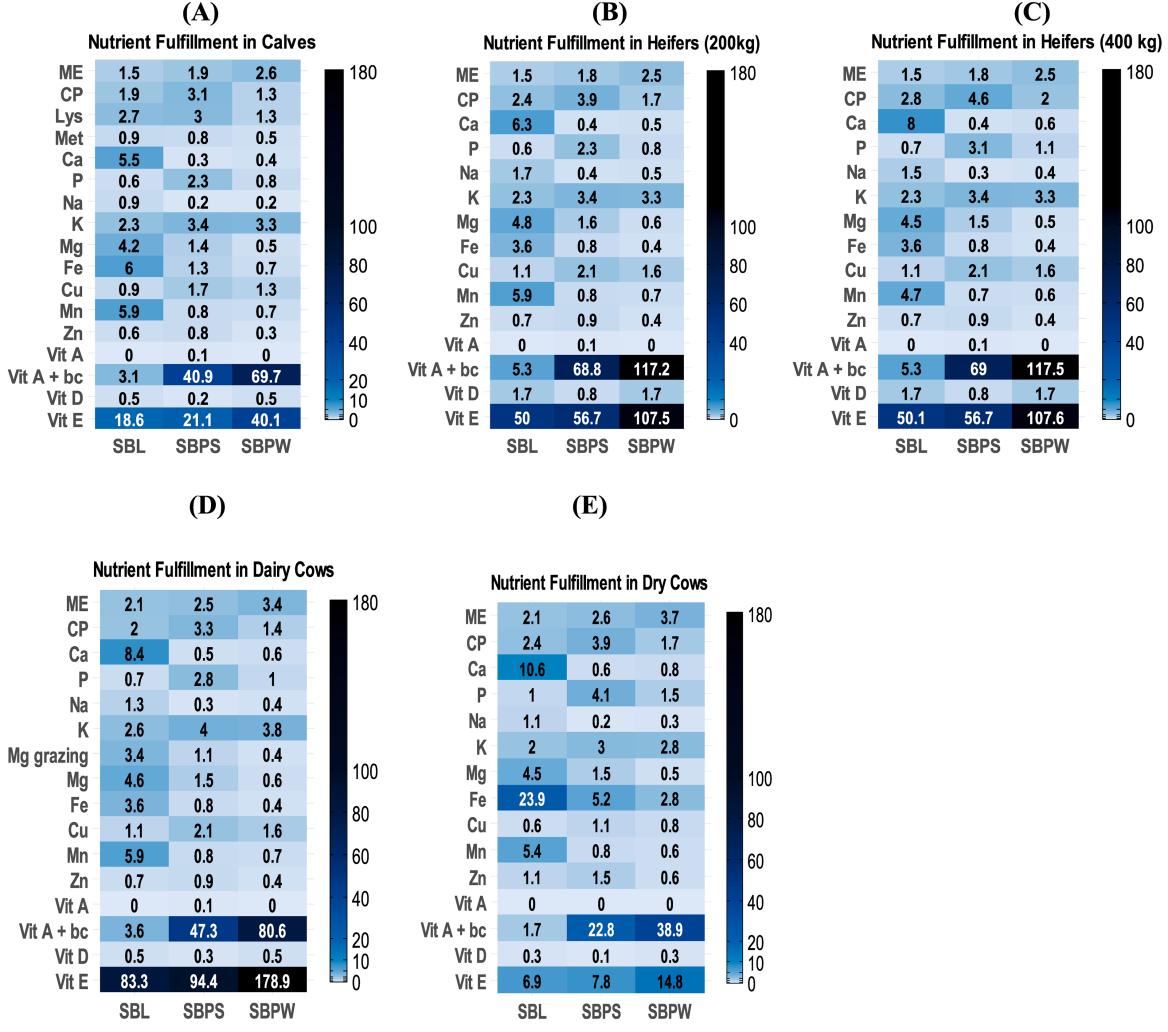
Percentage fulfillment of daily nutrient requirements in different categories of ruminants: calves **(A)**, heifers weighing 200 kg **(B)**, heifers weighing 400 kg **(C)**, dairy cows weighing 650 kg with 35 kg ECM **(D)**, and dry cows **(E)**, with 2.5% inclusion of sea buckthorn leaves (SBL), sea buckthorn pomace with seeds (SBPS), and sea buckthorn pomace without seeds (SBPW), in DMI. Interpretation: <2.5%: deficient; ≈2.5%: adequate; >2.5% rich or, if substantially higher, excessive. ME = metabolizable energy. CP = crude protein, Vit A + bc = vitamin A + hypothetical conversion of carotene to itamin A if we assume full conversion.

**Figure 4:**
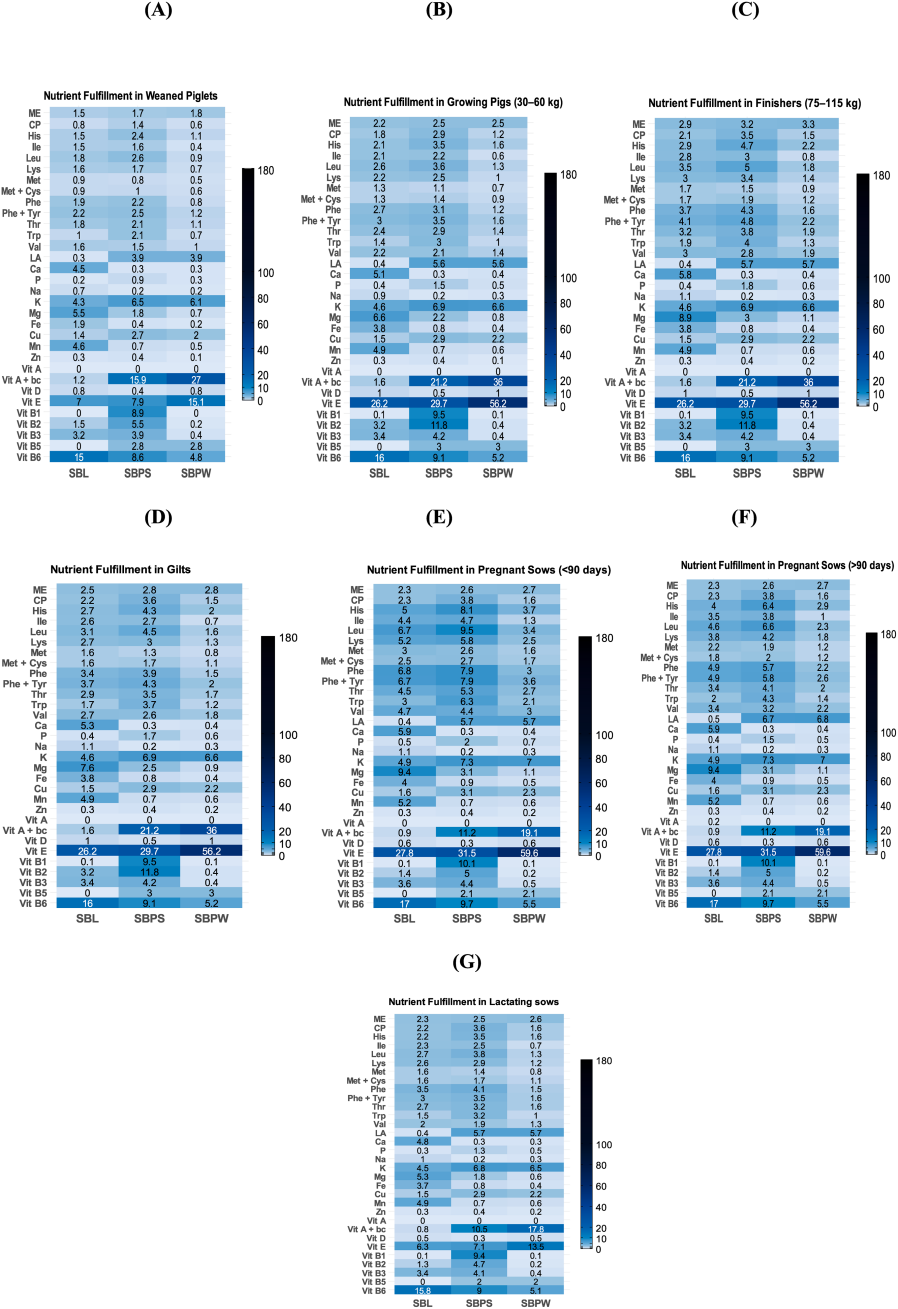
Percentage fulfillment of daily nutrient requirements in different categories of pigs: weaned piglets **(A)**, growing pigs weighing 30–60 kg **(B)**, finishers weighing 75–115 kg **(C)**, gilts **(D)**, pregnant sows (early gestation up to 90 days) **(E)**, pregnant sows (gestation after 90 days) **(F)**, and lactating sows **(G)**, with 2.5% inclusion of sea buckthorn leaves (SBL), sea buckthorn pomace with seeds (SBPS), and sea buckthorn pomace without seeds (SBPW), in DMI. Interpretation: <2.5%: deficient; ≈2.5%—adequate; >2.5%: rich or, if substantially higher, excessive. ME = metabolizable energy. CP = crude protein, Vit A + bc = vitamin A + hypothetical conversion of carotene to vitamin A if we assume full conversion.

**Figure 5:**
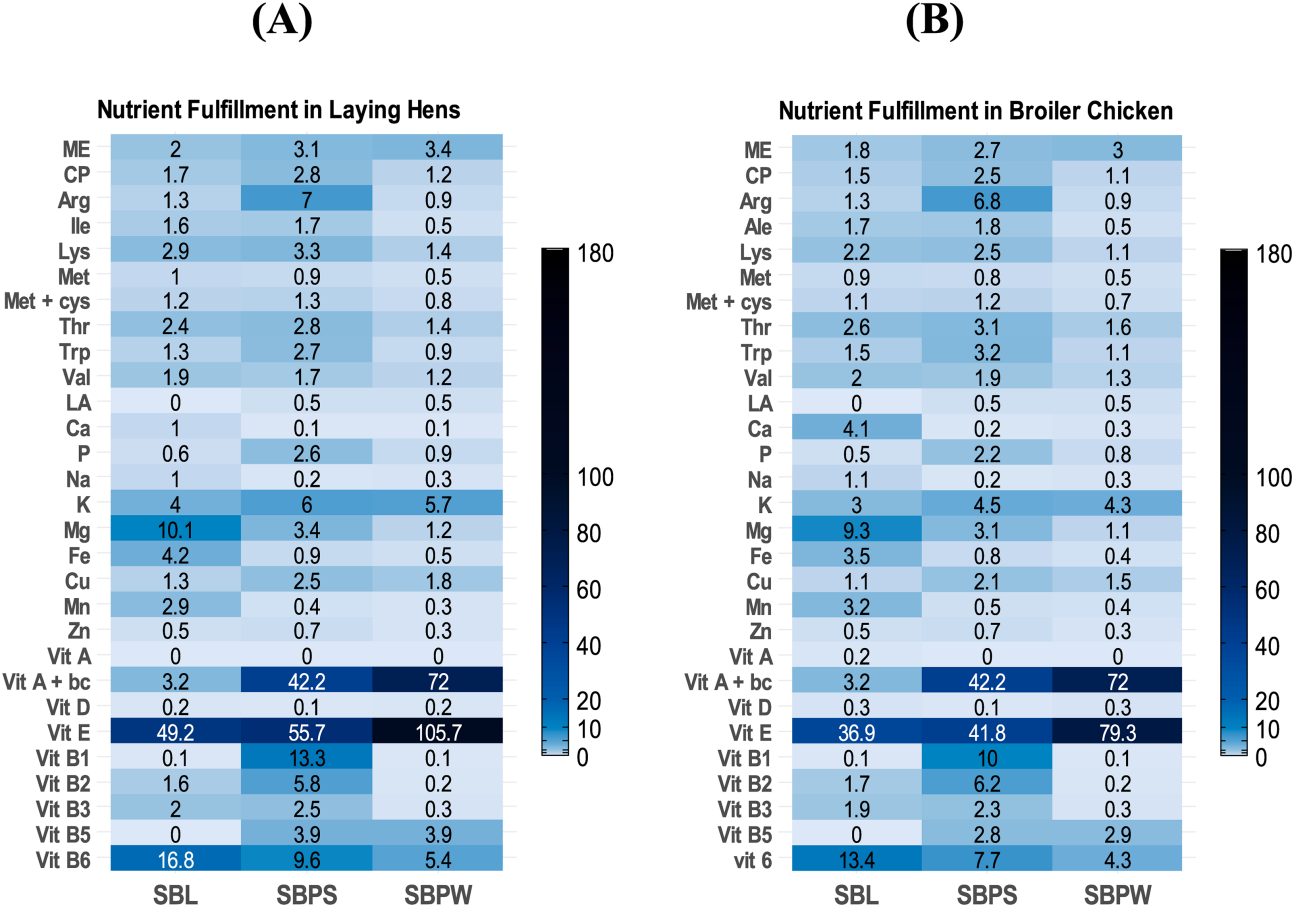
Percentage fulfillment of daily nutrient requirements in different categories of poultry: laying hens **(A)**, broiler chicken **(B)**, with 2.5% inclusion of sea buckthorn leaves (SBL), sea buckthorn pomace with seeds (SBPS), and sea buckthorn pomace without seeds (SBPW), in DMI. Interpretation: <2.5%: deficient; ≈2.5%—adequate; >2.5% rich or, if substantially higher, excessive. ME = metabolizable energy. CP = crude protein, Vit A + bc = vitamin A + hypothetical conversion of carotene to vitamin A if we assume full conversion.

Proximate analysis data are presented in Table 1, with a spider chart (Figure 1A) showing relative variation among samples. The presence of seeds in SBP significantly increased the overall CP content, with SBPS CP reaching nearly 225 g/kg DM compared to 96 g/kg DM in SBPW. Furthermore, SBPW exhibited the highest EE content, with fat levels notably exceeding those of SBPS. On a DM basis, seeds appear to contribute more protein than fat. The fat fraction of SBL was low compared to both pomaces, only 29 g/kg DM. Meanwhile, CF was highest in SBPS and lowest in SBL, consistent with NDF concentrations. ADF levels mirrored NDF values, with concentrations approximately 10% lower. Additionally, ingredients showed elevated ADIP levels. A clear difference was observed between the SBP fractions: ADIP accounted for 12.9% of CP in SBPW but 7.0% in SBPS. Regarding the energy contents of the SB residues, SBPW consistently exhibited the highest energy levels across both GE and ME for all animal species. GE values were similar for SBPW and SBPS, with SBPS slightly lower. Predicted ME varied among the by-products and also across animal species, indicating species-specific differences in energy utilization: SBL and SBPS were similar for horses and ruminants, whereas in broilers, ME of SBL was markedly lower than that of SBPS. Figures 2A–C, Figures 3A–E, Figure 4A–G and Figures 5A,B shows how SB residues fulfill the daily ME and CP requirements of different animal species and categories. SBPS contributed the most to CP requirements, whereas SBPW provided the largest proportion of daily ME across the animal categories.

**Table 1:** Proximate composition (average ± SD) of sea buckthorn leaves (SBL), sea buckthorn pomace with seeds (SBPS) and sea buckthorn pomace without seeds (SBPW).

Analyzed component	SBL *n* = 3	SBPS *n* = 3	SBPW *n* = 3
Dry Matter, g/kg DM	934 ± 9.0^a^	922 ± 6.6^a^	928 ± 12.9^a^
Organic Matter, g/kg DM	942 ± 0.7^b^	979 ± 2.1^a^	980 ± 0.5^a^
Crude Protein, g/kg DM	136 ± 4.4^b^	225 ± 4.2^a^	96 ± 2.8^c^
Crude ash, g/kg DM	58 ± 6.8^a^	21 ± 2.1^b^	20 ± 0.5 ^b^
Crude Fiber g/kg DM	143 ± 17.4^a^	189 ± 24.2^a^	154 ± 22.7 ^a^
Ether extract, g/kg DM	29 ± 9.4^b^	152 ± 33.8^a^	201 ± 20.1^a^
Nitrogen Free Extractives, g/kg DM	634 ± 24.4^a^	413 ± 4.5^c^	530 ± 7.3^b^
Neutral Detergent Fiber, g/kg DM	247 ± 0.9^b^	358 ± 18.4^a^	326 ± 17.3^a^
Acid Detergent Fiber, g/kg DM	210 ± 8.3^b^	291 ± 14.9^a^	267 ± 28.4^a^
Starch, g/kg DM	7 ± 0.1^a^	3 ± 0.2^b^	2 ± 0.1^c^
ADIP, % CP^1^	18.3	7	12.9
Assumed digestible CP, g/kg DM^1^	111.1	209.1	83.4
GE, MJ/ kg DM^2^	18.4	22.5	22.7
ME pigs (MJ/kg DM)^2^	13.6	15.1	15.6
ME broilers (MJ/kg DM)^2^	10.1	15.4	17
ME horses (MJ/kg DM)^2^	10.9	13	15.6
ME ruminants (MJ/kg DM)^2^	8.1	9.7	13.7

Values within a column with different superscript letters are significantly different (Tukey’s HSD test, *p* < 0.05).

1 ADIP (% of CP) and assumed digestible crude protein (g/kg DM) were calculated using mean values of ADF-CP, ADF, and crude protein. Therefore, only a single calculated value is reported and no standard deviation is available.

2 Metabolizable energy (ME; species-specific) and gross energy (GE) were calculated using mean values of chemical composition. Therefore, only single calculated values are reported and no standard deviation is available.

### 3.2 Mineral elements

**Figure 6:**
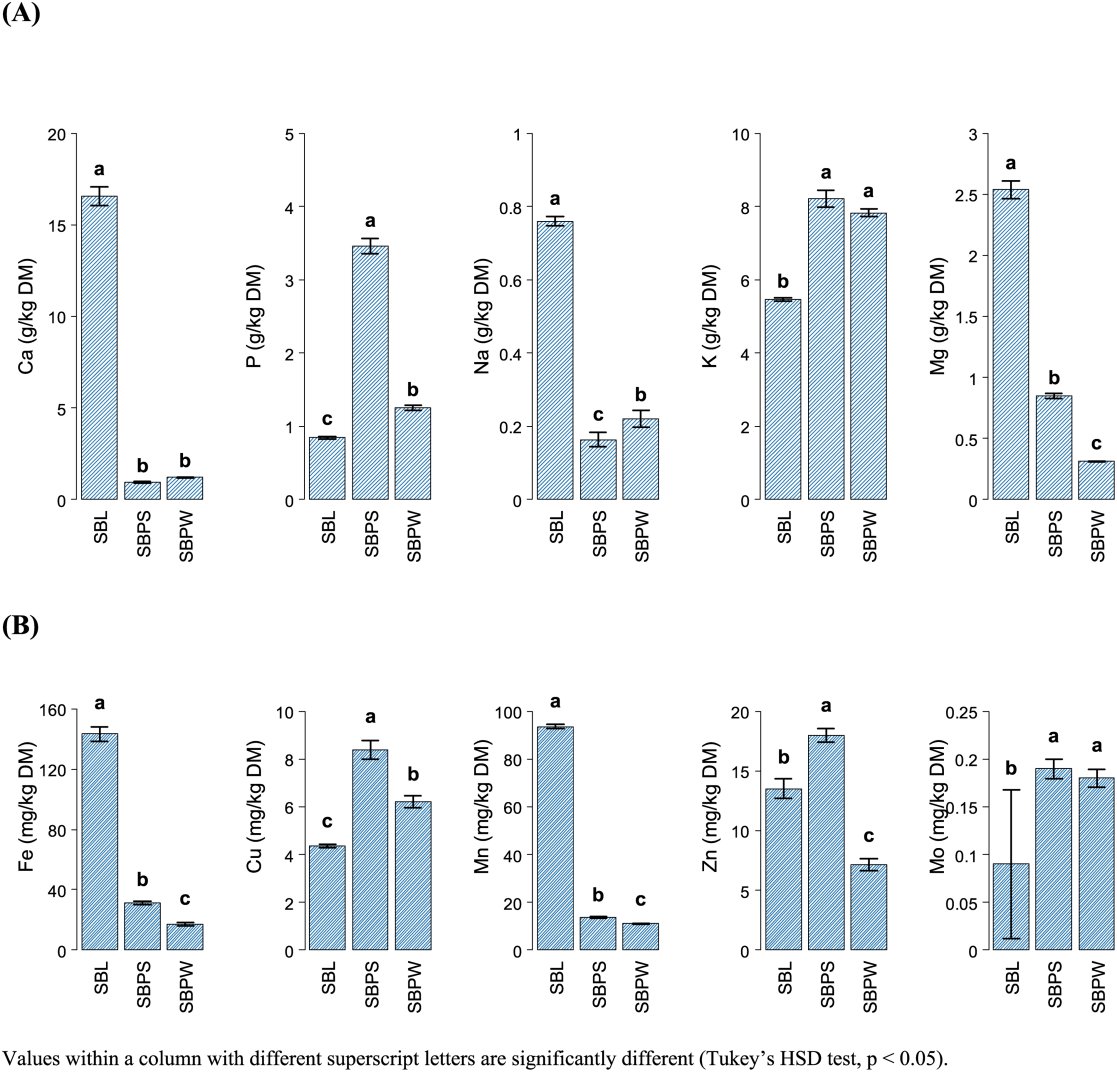
Content of macroelements **(A)** and microelements **(B)** of sea buckthorn leaves (SBL), sea buckthorn pomace with seeds (SBPS), and sea buckthorn pomace without seeds (SBPW) (*n* = 3). Values within a column with different superscript letters are significantly different (Tukey’s HSD test, *p* < 0.05).

As shown in Figures 1B, 6, illustrating a variation in mineral composition of samples, SBPW and SBPS had low levels of Ca, Na and Mg, compared to SBL. K levels were consistently high across all samples, with SBPW having the highest levels. Regarding microelements, the most pronounced differences were observed in Fe and Mn concentrations: SBL had nearly double the Fe content compared to both pomaces, with values as high as 143.3 mg/kg DM. More strikingly, Mn levels in SBL were approximately 7–8 times higher than those in both SBPW and SBPS, highlighting a significant disparity in microelement accumulation between these plant parts. Inclusion of seeds in the pomace increased the amounts of P, Mg, Zn, Cu, and Mn. The biggest difference between SBPW and SBPS was in the Zn and P concentrations. Figures 2A–C, Figures 3A–E, Figure 4A–G and Figures 5A,B illustrate SB residue’s contribution to fulfilling daily mineral requirements at 2.5% DMI inclusion level in horses, ruminants, pigs and poultry. All three SB residues supplied more than 2.5% of the daily K requirements across all animal species and categories. In the case of SBL, contributions also exceeded 2.5% of the daily requirements for Ca, Fe, Mn, and Mg. Cu concentration in SBPS was close or met 2.5% of the daily requirements for most animals’ groups. The contributions of other minerals to animals’ daily requirements were below the expected 2.5%.

### 3.3 Amino acids

Among the amino acids identified in SB residues, aspartic and glutamic acid consistently showed the highest concentrations (Table 2; Figure 1C). Proline was present in high amounts in SBL. Additionally, SBPS contained exceptionally high levels of arginine. The highest amounts of essential and non-essential amino acids were found in SBPS, and the lowest in SBPW, reflecting their CP content. Regarding other essential amino acids, leucine and lysine were consistently high among all three SB residues, while methionine and tryptophan were the most deficient, indicating that they are limiting in these by-products. Fulfillment of daily amino acid requirements, at 2.5% DMI inclusion for all three residues can be seen in Figures 2A–C, Figure 3A, Figures 4A–G and Figures 5A,B.

**Table 2:** Amino acid profile (average ± SD) of sea buckthorn leaves (SBL), sea buckthorn pomace with seeds (SBPS) and sea buckthorn pomace without seeds (SBPW) expressed as g/kg DM.

Amino acid	SBL (*n* = 3)	SBPS (*n* = 3)	SBPW (*n* = 4)
Essential amino acids			
Histidine (E)	3.5 ± 0.07^b^	5.6 ± 0.12^a^	2.5 ± 0.06^c^
Arginine (CE)^1^	6.0 ± 0.43^b^	31.4 ± 2.35^a^	4.1 ± 0.11^b^
Threonine (E)	7.2 ± 0.46^b^	8.5 ± 0.59^a^	4.3 ± 0.13^c^
Methionine (E)	1.7 ± 0.06^a^	1.4 ± 0.08^b^	0.9 ± 0.06^c^
Valine (E)	6.9 ± 0.47^a^	6.4 ± 0.26^a^	4.4 ± 0.20^b^
Tryptophan (E)	1.1 ± 0.16^b^	2.3 ± 0.17^a^	0.8 ± 0.04^c^
Phenylalanine (E)	7.6 ± 0.45^b^	8.8 ± 0.47^a^	3.3 ± 0.12^c^
Isoleucine (E)	5.1 ± 0.35^a^	5.4 ± 0.31^a^	1.5 ± 0.13^b^
Leucine (E)	11.9 ± 0.65^b^	16.8 ± 0.91^a^	5.9 ± 0.28^c^
Lysine (E)	10.2 ± 0.57^a^	11.3 ± 0.79^a^	4.8 ± 0.31^b^
Non-essential amino acids			
Serine (NE/CE)^2^	8.9 ± 0.65^b^	15.9 ± 1.09^a^	4.4 ± 0.15^c^
Aspartic acid (NE)	19.9 ± 1.43^b^	34.6 ± 2.55^a^	20.6 ± 0.26^b^
Glutamic acid (NE)	13.0 ± 1.25^c^	39.0 ± 5.22^a^	19.6 ± 0.58^b^
Glycine (NE/CE)^3^	7.7 ± 0.54^b^	12.0 ± 0.93^a^	4.6 ± 0.15^c^
Alanine (NE)	7.4 ± 0.28^b^	10.7 ± 0.78^a^	4.5 ± 0.11^c^
Proline (NE/CE)^4^	13.8 ± 0.75^a^	9.2 ± 0.36^b^	3.6 ± 0.24^c^
Tyrosine (NE/CE)^5^	5.8 ± 0.38^b^	6.9 ± 0.42^a^	3.8 ± 0.10^c^
Cysteine (NE/CE)^6^	1.7 ± 0.22^b^	2.3 ± 0.09^a^	1.5 ± 0.11^b^

Values within a column with different superscript letters are significantly different (Tukey’s HSD test, *p* < 0.05).

1 Arginine is essential for pigs and poultry and conditionally essential for horses and young animals. It is not considered essential for cattle.

2 Serine is conditionally essential in young or rapidly growing animals.

3 Glycine is semi-essential for young or rapidly growing pigs and young poultry.

4 Proline is conditionally essential (CE) for young or rapidly growing animals, particularly foals and piglets.

5 Tyrosine is conditionally essential when phenylalanine is limiting.

6 Cysteine is conditionally essential when methionine is limiting.

### 3.4 Fatty acids

Palmitic and palmitoleic acids were present at relatively high proportions in all three residues, but levels were significantly lower in SBL—due to its lower fat content—and considerably higher in SBPW (Table 3). Additionally, all residues proved to be notable sources of linoleic acid (LA), an omega-6 (n-6) fatty acid, with comparable concentrations in both SBPW and SBPS. In contrast, α-linolenic acid (ALA), an omega-3 (n-3) fatty acid, was higher in SBPS compared to SBPW. Palmitic acid was the most prominent fatty acid in SBL, palmitoleic acid in SBPW, and LA in SBPS.

**Table 3:** Fatty acid composition (average ± SD) of sea buckthorn leaves (SBL), sea buckthorn pomace with seeds (SBPS) and sea buckthorn pomace without seeds (SBPW), expressed as g/kg DM.

Fatty acid	SBL *n* = 3	SBPS *n* = 3	SBPW *n* = 3
C14:0	0.04 ± 0.003^b^	0.04 ± 0.001^b^	0.11 ± 0.007^a^
C15:0	0.01 ± 0.001^a^	0.01 ± 0.006^a^	0.01 ± 0.008^a^
C16:0	0.57 ± 0.032^c^	2.08 ± 0.072^b^	5.82 ± 0.719^a^
C16:1c7	0.03 ± 0.000^a^	0.00 ± 0.001^b^	0.01 ± 0.003^b^
C16:1c9	0.17 ± 0.003^c^	1.75 ± 0.079^b^	6.00 ± 0.731^a^
C17:0	0.00 ± 0.002^a^	0.00 ± 0.000 ^a^	0.00 ± 0.000 ^a^
C17:1c10	0.00 ± 0.001^c^	0.05 ± 0.045 ^b^	0.15 ± 0.128 ^a^
C18:0	0.05 ± 0.003^c^	0.18 ± 0.007^a^	0.16 ± 0.003^b^
C18:1c9	0.15 ± 0.011^b^	0.89 ± 0.043^a^	0.85 ± 0.145^a^
C18:1c11	0.06 ± 0.004^c^	0.44 ± 0.010^b^	1.04 ± 0.093^a^
C18:2c/t+	0.01 ± 0.007^a^	0.01 ± 0.005^a^	0.01 ± 0.007^a^
C18:2n6, Essential fatty acid	0.17 ± 0.009^b^	2.46 ± 0.191^a^	2.48 ± 0.088^a^
C18:3n6	0.00 ± 0.000	0.00 ± 0.000	0.00 ± 0.000
C20:0	0.05 ± 0.001^a^	0.04 ± 0.000^b^	0.04 ± 0.005^a^
C18:3n3, Essential fatty acid	0.20 ± 0.031^b^	1.39 ± 0.178^a^	0.31 ± 0.019^b^
C20:1c11	0.01 ± 0.003^b^	0.02 ± 0.007^a^	0.03 ± 0.007^a^
C18:4n3	0.01 ± 0.006^a^	0.00 ± 0.002^a^	0.01 ± 0.011 ^a^
C21:0	0.00 ± 0.000	0.00 ± 0.000	0.00 ± 0.000
C20:2n6	0.00 ± 0.000^a^	0.01 ± 0.003^a^	0.02 ± 0.009 ^a^
C22:0	0.11 ± 0.002^a^	0.02 ± 0.003^c^	0.04 ± 0.001^b^
C23:0	0.02 ± 0.018^a^	0.00 ± 0.004 ^a^	0.00 ± 0.003 ^a^
C24:0	0.04 ± 0.001^a^	0.02 ± 0.004^b^	0.04 ± 0.003^a^
SFA, %	52.38 ± 1.95	25.74 ± 0.159	36.25 ± 0.383
MUFA, %	24.61 ± 0.25	33.72 ± 0.708	47.14 ± 0.988
PUFA	23.01 ± 2.20	40.87 ± 1.414	16.61 ± 1.369
Total trans	0.74 ± 0.02	0.10 ± 0.003	0.07 ± 0.012
n6, %	10.28 ± 0.83	26.06 ± 0.377	14.65 ± 1.127
n3, %	12.24 ± 1.80	14.73 ± 1.077	1.91 ± 0.276
n6/n3 ratio	0.85 ± 0.05	1.77 ± 0.100	7.71 ± 0.496

Values within a column with different superscript letters are significantly different (Tukey’s HSD test, *p* < 0.05).

C14:0—myristic acid; C15:0—pentadecylic acid; C16:0—palmitic acid; C16:1c7—cis-7-hexadecenoic acid; C16:1c9—palmitoleic acid, cis-9-hexadecanoic acid; C17:0—margaric acid; C17:1c10—Cis-10 heptadecenoic; C18.0—stearic acid; C18:1c9—oleic acid, trans-9; C18:1c11—vaccenic acid; C18:2c/t+—Total linoleic-type fatty acids (cis + trans isomers combined); C18:2n6—linoleic acid; C18:3n6—gamma-linolenic acid, C20:0—arachidic acid; C18:3n3—alpha-linolenic acid; C20:1c11—Gondoic acid; C18:4n3-stearidonic acid; C21:0 heneicosanoic acid; C20:2n6—eicosadienoic acid; C22—behenic acid; C23:0—tricosanoic acid; C24:0—lignoceric acid.

SBL had a high proportion of saturated fatty acids (SFA). In contrast, both pomaces contained higher proportions of unsaturated fatty acids: monounsaturated (MUFA) and polyunsaturated fatty acids (PUFA) than SFAs. Regarding the n6:n3 ratio, SBPW had the highest proportion of n6 fatty acids, with a ratio of 7.7:1. In contrast, SBL had the lowest proportion of n6 fatty acids compared to n3, resulting in a ratio below 1. For SBPS, the ratio was 1.77:1, indicating a slightly higher proportion of n6 than n3 fatty acids. Regarding the fulfillment of daily LA requirements in pig categories, at 2.5% DMI inclusion, SBPW and SBPS exceeded the expected 2.5% contribution, whereas SBL did not meet this level (Figure 4A–G). In poultry, however, all three residues supplied less than 2.5% of the daily LA requirement (Figures 5A,B).

### 3.5 Vitamins

All three SB residues had high concentrations of vitamin E, with SBPW containing the highest levels (Table 4). Moreover, both pomaces showed higher β-carotene contents, consistent with their characteristic orange pigmentation. However, SBPS had lower β-carotene levels than SBPW, likely due to dilution by the presence of the seeds. The results also indicate that seeds contribute to higher concentrations of vitamins B_1_, B_2_, B_3_, and B_6_, compared to SBPW. While all three residues exhibited high pyridoxine concentrations, the highest levels were observed in SBL. In contrast, vitamin B_5_ was not detected in SBL. When it comes to fulfillment of daily requirements, the contribution of SB residues’ vitamin content to animals’ daily requirements is presented in Figures 2A–C, Figures 3A–E, Figure 4A–G and Figures 5A,B. The combined content of vitamin A and β-carotene, assuming complete conversion of β-carotene to vitamin A, in all three SB residues substantially exceeded the daily vitamin A requirements of all categories of horses, ruminants (except SBL’s content of vitamin A and β-carotene in dry cows, Figure 3E), pigs (except SBL where the contribution of the combined content of vitamin A and β-carotene, was below 2.5% of daily requirements) and poultry. Similarly, due to the high vitamin E concentrations in all three SB materials, the set 2.5% of daily requirements was considerably exceeded across all animal categories. In SBPS, all B vitamins (B_1_–B_6_) supplied more than 2.5% of the daily requirements across species, while pyridoxine (vitamin B_6_) concentrations of all studied SB residues contributed more than 2.5% of the animals’ daily needs. In SBL, riboflavin (vitamin B_2_) and niacin (vitamin B_3_) met or were close to the 2.5% requirement contribution threshold.

**Table 4:** Vitamin profile of sea buckthorn leaves (SBL), sea buckthorn pomace with seeds (SBPS) and sea buckthorn pomace without seeds (SBPW), expressed as mg/kg DM.

Vitamin	SBL *n* = 1	SBPS *n* = 1	SBPW *n* = 1
Vitamin A, retinol	<0.02^1^^,^^2^	<0.05^1^^,^^2^	<0.02^1^^,^^2^
β-carotene	11.5	151.3	257.9
Vitamin D, tocopherol	<0.01^1^^,^^3^	<0.005^1^^,^^3^	<0.01^1^^,^^3^
Vitamin E, calciferol	335.4	378.0	720.5
Vitamin B_1_, thiamine	<0.1^1^^,^^4^	9.08	<0.1^1^^,^^4^
Vitamin B_2_, riboflavin	3.1	11.2	0.4
Vitamin B_3_, niacin	32.5	39.7	4.3
Vitamin B_5_, pantothenic acid	0.0	14.2	14.3
Vitamin B_6_, pyridoxine	22.9	13.1	7.4

1 Values shown as “<” are below the analytical limit of detection (LOD).

Vitamin concentration below the limit were not converted to mg/kg DM but remained as mg/kg.

2 For vitamin A, the LOD was 0.01 μg/g for all samples except SBPS (0.05 μg/g).

3 For vitamin D, the LOD was 0.01 μg/g for all samples except SBPS (0.005 μg/g), as SBPS was measured later.

4 The LOD for vitamin B_1_ was 0.1 μg/g for all samples.

## 4 Discussion

### 4.1 Proximate analyses

Proximate analyses provide fundamental information on the nutritional profile of a feed ingredient. In this study, proximate composition of SB residues differed from previous reports (17, 56, 57) and varied markedly among materials, with the highest fat levels in SBPW, the highest crude protein in SBPS, and the lowest organic matter observed in SBL. These compositional differences suggest that each residue offers distinct nutritional advantages, SBPW as an energy source, SBPS as a protein supplement, and SBL as a mineral-rich ingredient.

Although SBPS had higher CP content compared to SBPW, indicating that seed fraction contributes considerably to the protein content, whole seeds may pass undigested through digestive tract and require grinding for improved nutrient availability, much like cereal grains (58, 59). SBL’s protein composition aligned with expectations for foliage. Despite differences observed in protein content, all three materials fulfilled the required minimal CP amount of 70 g/kg DM for microbial protein synthesis according to Van Soest (60) and the NRC (61). SBPS exceeded 2.5% of the daily protein requirements for most animal species, whereas SBPW contributed the least, indicating that it would need to be carefully balanced with high-protein ingredients. However, high ADIP values, found in all three SB residues, suggest that a portion of protein in these by-products is likely bound to fiber or altered by Maillard reactions, reducing its digestibility and amino acid bioavailability. Such interactions can limit the effective contribution of these materials to dietary protein supply, highlighting the need to consider both processing conditions and complementary protein sources in feed formulation. Moreover, SBPS showed lower ADIP values compared to SBPW, suggesting that the seed fraction of the material contains higher-quality protein. ADIP concentrations have not previously been reported in sea buckthorn leaves and pomaces, making the comparison with our results difficult. Still, interpretation of ADIP should be done carefully, as this method can both over or underestimate protein digestibility. In non-forage, heat processed feed materials, ADIP has been reported to overestimate protein indigestibility, as results have shown that much of the protein fraction considered indigestible was in fact rumen degradable (62, 63). Discrepancies can also arise during the analytical process, such as nitrogen contamination from Cetrimonium bromide during ADF analysis (64). It is important to note that ADIP in SB residues was higher than typically reported for conventional feed ingredients as reported by Mc Donald et al. (37) (Supplementary Table S8).

In relation to lipid composition, EE content was higher in SBPW than in SBPS, contrary to expectations that inclusion of seeds would boost fat levels. This outcome is likely a result of carotenoid-rich lipids present in the pomace fraction of SB (65).

High CF observed in residues is especially relevant for poultry and weaned piglets as these species have a limited ability to digest fiber, and excess fiber levels in their diet might result in reduced nutrient availability. At the same time, dietary fiber offers benefits in these species: soluble fiber supports gut health and the maintenance of the intestinal barrier, while insoluble fiber increases fecal bulk, aiding stool management (66). High CF diets are associated with different positive metabolic effects and a diverse, healthy microbiota (67). Additionally, older categories of pigs have a greater ability to digest fiber. Despite this capacity remaining limited, high-fiber diets in group-housed gestating sows increase the feeling of satiety, reduce the hunger and therefore decrease aggressive and stereotypic behavior (68). Accordingly, high-CF ingredients have been progressively included in the diets of these animals, although careful control of their intake remains necessary. Apart from CF, SB residues showed high levels of NFE- indicating that carbohydrates constitute the major component of the DM of these samples. Carbohydrates play essential roles in animal metabolism–monosaccharides provide immediate energy while polysaccharides serve as structural components in plant cell walls and as energy storage in plant tissues (57). Starch content was very low in SBP, which is consistent with findings from previous studies, likely due to juice extraction and naturally low starch in ripe fruits (69–71). SBL also exhibited very low starch concentrations. Starch content has not been previously evaluated in SBL, making direct comparison with earlier studies impossible. Further research into the carbohydrate composition in SB by-products is required.

ME values of SB residues varied across species and the by-products themselves due to differences in digestive physiology of animals and by-products’ composition. SBPW provided a relatively higher energy contribution compared to the other two residues and many conventional feed ingredients, particularly in broilers (Supplementary Table S7). It met the proportional 2.5% daily ME requirements across most animal species, except weaned piglets, as can be seen in Figures 2A–C, Figures 3A–E, Figure 4A–G and Figures 5A,B, whereas SBL contributed the least. However, it is important to note that the reported ME values have limitations. Fiber utilization in pigs improves with body weight, so in general it is advisable to report separate ME values of feeds for growing and adult pigs (72). However, this was not applied in this study, possibly causing under- or overestimation of ME in certain pig categories. Moreover, the AMEn prediction equation was originally developed for male broilers, therefore, applying it for laying hens might be unreliable. Lastly, all obtained ME values were acquired using prediction equations and may not reflect the material’s true species-specific energy contents. Future research is necessary to acquire more accurate values. In light of these factors, it is important to interpret the reported ME values carefully.

### 4.2 Minerals

Mineral analyses are important to evaluate the nutritional value, safety, and suitability of materials as feed sources. In the present investigation, the mineral composition of SB residues differed notably from earlier reports (73, 74). Overall, none of the residues supplied adequate Na or Zn at a 2.5% DMI inclusion rate, while K was consistently high and Ca, Mg, and P varied sharply among materials. These patterns suggest that although SB residues can complement certain minerals, they cannot serve as primary mineral sources and must be balanced within complete diets.

Across all three residues, Na and Zn contributed less than 2.5% of daily requirements. Although Na and Zn deficiencies are unlikely in practical diets due to routine supplementation, it is noteworthy that Zn concentrations in the residues were lower than in conventional feed ingredients such as cereals, legumes, oilseed by-products and roughages (Supplementary Table S9) (44, 45).

Potassium concentrations were relatively high and exceeded 2.5% of daily K requirements in most species at a 2.5% inclusion rate. However, SB potassium levels were still lower than in most forages and oilseed by-products and comparable to cereals (Supplementary Table S9), making excessive K intake unlikely. Nonetheless, high dietary K can interfere with Mg and Ca absorption, and especially in ruminants and transition cows, can predispose animals to hypocalcemia and hypomagnesemia (75, 76). High K intake also affects Na metabolism. Thus, total dietary K load should be considered when SB residues are included in diets.

Both pomaces were low in Mg, particularly SBPW, which contained less Mg than any conventional feed ingredient such as forages, cereals, etc., whereas SBL proved to be a comparatively good Mg source (Supplementary Table S9). Ca concentrations in SBL were also high and comparable to legumes, meeting or exceeding 2.5% of daily Ca requirements in most species at a 2.5% inclusion rate (Supplementary Table S9; Figures 2A–C; Figures 3A–E; Figure 4A–G; Figures 5A,B). Laying hens were the exception due to their exceptionally high Ca demand for eggshell formation (Figure 5A). In contrast, Ca levels in SBPW and SBPS were low, similar to cereals, and insufficient for most species (Supplementary Table S9). Phosphorus concentrations were deficient in SBPW and SBL and adequate or only slightly deficient in SBPS for most species (Figures 2A–C; Figures 3A–E; Figure 4A–G; Figures 5A,B). However, these estimates do not account for the low bioavailability of P in monogastrics, as most plant-derived P exists as phytate, which is largely indigestible (77, 78). Even in ruminants, microbial phytase does not completely degrade phytate, reducing P absorption (76). Phytate also binds Ca, Fe, and Zn, further lowering their bioavailability in monogastrics unless exogenous phytase is supplemented (79). In addition, maintaining an appropriate Ca:P ratio is essential across species, as imbalances can impair bone development, reduce growth, and cause metabolic disorders (76, 80).

SBL also contained elevated Fe and Mn levels, exceeding 2.5% of daily requirements for most species at 2.5% inclusion (Figures 2A–C; Figures 3A–E; Figure 4A–G; Figures 5A,B). Weaned piglets were an exception, as their Fe needs are unusually high due to low Fe reserves at birth, the very low Fe content of sow milk, and the demands of large litters (Figure 4A) (81). SBPW and SBPS contained substantially lower Fe and Mn, more closely resembling conventional cereals (Supplementary Table S9). All SB residues at 2.5% inclusion failed to meet 2.5% of daily Cu requirements for horses and ruminants, although SBPS approached the threshold in some categories (Figures 2A–C; Figures 3A–E). In pigs and laying hens, Cu concentrations in SBPS, and to a lesser extent SBPW, approached or exceeded daily needs (Figure 4A–G; Figure 5A). However, Cu adequacy or toxicity risk depends more on bioavailability than on total concentration, as absorption is strongly reduced by antagonists such as sulfur, molybdenum, and iron (82, 83). In the context of SB residues, Mo levels were very low and would not be expected to inhibit Cu absorption, although the high Fe content of SBL could reduce Cu utilization if fed at higher inclusion rates. Importantly, mineral antagonisms arise from the total dietary composition rather than a single ingredient; thus, overall diet formulation must consider the cumulative balance of interacting minerals.

### 4.3 Amino acids

Amino acid (AA) analysis provides insight into the protein quality and biological value of feed materials beyond crude protein content, as AA are essential for growth, reproduction and immune and metabolic processes in animals (84, 85). In this study, AA profiles of SB residues showed little variations compared with previous reports (11, 57), though higher methionine and lower proline were previously noted. Methionine was consistently low in SB residues. In fact, it was the first limiting amino acid in SBPS, while tryptophan was in SBL and SBPW. SBPW supplied insufficient essential AAs for most animal categories, particularly poultry. In contrast, SBL and SBPS exhibited more balanced AA profiles, in fact, in pregnant sows, all essential AAs met or exceeded 2.5% of their daily requirements (Figures 4E,F). In weaned piglets, SB residues contributed the least to AA daily requirements (Figure 4A). SBPS was particularly rich in arginine, serine, and aspartic acid, while SBL contained high proline concentrations. Across all three SB residues, histidine, phenylalanine, leucine, phenylalanine + tyrosine and threonine contributed most to the daily amino acid requirements in all of the studied animal species and categories (Figures 2A–C, Figures 3A–E, Figure 4A–G and Figures 5A,B).

Methionine deficiency may impair sulfur and methyl group metabolism, antioxidant balance, and protein synthesis (86). Its supplementation would be required if these materials were to be incorporated into animals’ diets. The high arginine content in SBPS, an essential amino acid for weaned piglets and poultry, could support growth, immune function, and reproduction of animals (87–89). Additionally, high serine and aspartic acids, also observed in SBPS, could contribute to antioxidant defense, intestinal integrity, and energy metabolism (90–92). Due to their antioxidant properties, these AAs could benefit animals exposed to oxidative challenges, (such as during transport, weaning, pregnancy, vaccination, or disease) as well as animals fed low-protein diets. However, since both serine and aspartic acid are non-essential amino acids, that can be synthesized endogenously, the nutritional advantage of their high concentrations in SBPS is limited. Meanwhile, SBL exhibited high proline concentrations, even when compared to other protein-rich ingredients such as oilseed by-products (Supplementary Table S10). Proline supports collagen formation, intestinal growth, and oxidative protection (93, 94). It is conditionally essential for weaned piglets and may also benefit animals under metabolic or oxidative strain (95, 96).

SBL and SBPS could serve as supplementary protein sources for pigs and other livestock, although methionine and tryptophan supplementation would be required. However, inclusion rates may be limited by fiber or palatability. Evaluating performance responses *in vivo* would clarify whether its apparent amino acid balance translates into improved nitrogen efficiency and growth. The balanced AA composition of SBPS relative to its CP, even compared with higher-protein ingredients such as oilseed by-products, suggests potential for inclusion of this by-product in reduced-protein diets aimed at minimizing nitrogen excretion without compromising performance (Supplementary Table S10). In case of SBPW, amino acid supplementation would be needed to maintain protein turnover and antioxidant protection, particularly in the fast-growing animals.

However, fulfillment of amino acid daily requirements, should be interpreted carefully, as they assume 100% digestibility and neglect matrix effects, while in practice amino acid availability might be considerably reduced, depending on the species, processing conditions and presence of antinutritional compounds (97, 98). Just based on ADIP content, AA availability of these residues is expected to be substantially reduced. Actual AA bioavailability and absorption in different animal species and categories warrant further investigation. Moreover, while total daily amino acid requirements were used for pigs, the comparison for poultry was based on digestible amino acid daily requirements from McDonald et al. (37) which likely led to an overestimation of requirement fulfillment. When using Luke reference values, it was unclear whether these represented total or digestible amino acids, introducing additional uncertainty to the interpretation (46). Lastly, amino acid results obtained in this study are based on freeze-dried material. As freeze drying is known for preserving nutrients more effectively when compared with other drying methods, it is likely that observed AA values are higher than they would be in oven-dried samples (99). However, due to cost and sustainability considerations, freeze drying is unlikely to be used for feed materials. This provides an additional reason why the calculated fulfillment of daily amino acid requirements in this study may be overestimated and should be interpreted accordingly.

### 4.4 Fatty acids

Fatty acids represent concentrated energy sources with unsaturated forms typically associated with health-promoting effects. Fatty acid profiles largely matched previous reports, though some studies have found variations in alpha-linolenic acid (ALA), palmitic, and palmitoleic acid levels (11, 71, 100). In this study, SBPS contained the most PUFA, mainly ALA, exceeding those found in most cereals, oilseed by-products, and legumes. Linoleic acid (LA), on the other hand, was lower in SB residues than in the mentioned conventional feed ingredients (Supplementary Table S11). SBPW was richest in MUFA (dominated by palmitoleic and oleic acids), and SBL in SFA (dominated by palmitic acid). SBL showed the lowest n6:n3 ratio (0.85:1), whereas SBPW had the highest (7.71:1). These patterns indicate that SB residues differ markedly in fatty acid profiles and should be combined strategically to balance lipid quality in diets, with SBPS showing the most promise with a good n6:n3 and high PUFA concentrations.

The essential fatty acids LA and ALA are vital for reproduction, growth, and immune function (101, 102). A low n6:n3 ratio and higher n3 levels are generally associated with improved fertility, embryo survival, and beneficial fatty acid profiles in animal products such as milk, eggs, and meat (103, 104). In contrast, excessive SFAs can negatively affect plasma cholesterol, reproduction, and immune function (105). Consequently, both human and animal nutrition have shifted toward increasing dietary unsaturated fatty acids, including enriching animal products through feed supplementation. In this context, high concentrations of unsaturated FA in pomaces could enhance reproductive efficiency, product quality, and environmental sustainability by lowering enteric methane emissions (101, 106, 107). SBPW may serve as a functional source of MUFAs for improving product oxidative stability and animal performance, while SBL’s low lipid content limits its practical influence on dietary FA profiles (108). Oleic acid, abundant in all residues, is linked to reduced methane emissions in ruminants and improved cardiovascular and metabolic outcomes in humans (109–111). SB residues, especially SBPW, were also rich in palmitic acid, an important energy source that can improve milk yield and digestibility but may impair body condition when excessive (112, 113).

Together, SB residues could contribute to diversifying lipid sources in feed formulations, especially where improving n3 intake (SBPS) or product oxidative stability (SBPW) is desired. When assessing daily fatty acid requirement fulfillment in pigs at a 2.5% inclusion level of residues, SBPW and SBPS provided more than the proportional (2.5%) requirement of LA, while SBL contributed less than 2.5% (Figure 4A–G). However, the interpretation of requirement fulfillment is constrained by inconsistent reference values: NRC recommendations for LA are considerably lower than those observed in research trials (34, 101, 102, 114). For instance, NRC (34) recommends 6 g per day of LA for lactating sows and 1.5–2.8 g per day for growing pigs, whereas research studies have reported optimal levels of 125 and 15–30 g per day, respectively, showing that on farms linoleic acid is provided in much higher concentrations (101, 102, 114). Meanwhile, in both categories of poultry, all three residues failed to meet 2.5% of daily LA requirements, indicating a need for supplementation (Figures 5A,B). Species-specific FA metabolism and absorption efficiencies also require further investigation through controlled feeding experiments.

### 4.5 Vitamins

Vitamin analysis help identify feed ingredients that can supply essential vitamins which support animal health, performance, and ultimately contribute to reduction of reliance on synthetic supplementation. Previous research on vitamins in *Hippophae rhamnoides* has focused primarily on leaves and berries, with limited information on residues. However, some studies have analyzed tocopherols and β-carotene in pomace, showing varied results (11, 71). In this study, SB residues were consistently rich in vitamin E with pomaces particularly rich in β-carotene, exceeding levels typically found in conventional feed ingredients (Supplementary Table S12). Conversely, vitamin A and D levels were uniformly low, aligning with those of standard feedstuffs such as cereals (Supplementary Table S12). Results on vitamin B varied sharply among materials, with high content of these vitamins found in SBPS.

In some animal categories, addition of SB residues in the diet of animals could theoretically lead to exceeding of daily vitamin E and A requirements, if full conversion of β-carotene to vitamin A is assumed (Figures 2A–C, Figures 3A–E, Figure 4A–G and Figures 5A,B). High intakes of high vitamin A have been associated with signs of toxicity. For example, pigs fed excess vitamin A for 5 weeks developed lesions in endochondral and intramembranous bones (34). Toxicity of vitamin A in pigs typically presents with hyperirritability, crusty skin, coarse dull hair, haematuria, haematochezia, blood in the skin and hooves, periodic tremors, and loss of leg control (34). Feeding 500,000 IU/d of vitamin A to dry cows has been reported to reduce milk yields, while dose of 1,300 IU/kg BW has been found to cause osteoporosis (48). In horses, vitamin A toxicity has been linked to high bone fragility, exfoliated epithelium, hyperostosis, and teratogenesis (53). The safe upper limit of vitamin A, or Retinyl acetate, has been determined in an EU Directive for each animal species (115). In contrast, negative impacts of excess intakes of vitamin E or β-carotene have not been reported. Vitamin E and β-carotene play key antioxidant, reproductive, and immune roles in animals. High concentrations of vitamin E found in all three residues could especially be of benefit to gestating animals, especially cows in transition period, when demands of vitamin E increase due to its role in both reproductive health and immunity, but also for other animals exposed to stress or high metabolic demands (34, 48, 53). Bioavailability and storage degradation of vitamin E and β-carotene likely reduce the risk of exceeding requirements. In addition, not all ingested β-carotene is likely to be converted to vitamin A, making potential toxicity even more unlikely.

High levels of B-complex vitamins in SBPS, with pyridoxine concentrations elevated across all three residues, may support growth as well as energy and protein metabolism, potentially reducing the need for synthetic vitamin supplementation in nutrient-dense or high-protein, fast-growth diets (34, 48). Niacin levels were also high in both SBPW and SBL, although these concentrations are comparable to, or lower than, those found in some traditional feed ingredients (44) (Supplementary Table S12).

SB residues, especially SBPS, show promise as natural sources of antioxidants (vitamin E and β-carotene) and B-vitamins for livestock. Their inclusion could enhance vitamin supply, reduce reliance on synthetic premixes, and support animal performance and immune health, while contributing to the sustainability of feed formulations through the valorization of agro-industrial by-products.

Because vitamin analyses were not temporally aligned with other nutrient assays, degradation during storage may have biased inter-material comparisons. Future work should standardize sampling times or include storage-stability controls. Additionally, as described in the methodology, for vitamins below detection limits, half of the detection limit was used as an estimate to calculate daily requirement fulfillment. While practical, this substitution does not reflect exact vitamin concentrations, so percentages of daily requirements might be over or underestimated. Lastly, because vitamin measurements were based on a limited number of replicates, the vitamin-related findings should be interpreted as exploratory rather than definitive. Nevertheless, the clear differences observed among residues and alignment with previously published values provide confidence in the general patterns reported. Future studies should include additional replication to strengthen quantitative precision.

### 4.6 Summary of nutritional and practical observations and animal suitability

For most analyses—particularly mineral, vitamin, and proximate concentrations—previous studies reported results that differed from ours. These observed differences likely arise from factors such as SB variety, plant maturity, harvest time, processing, drying, storage, climatic conditions and inherent natural variability (116–118). Regarding animal category/species suitability, based on the proportion of daily nutrient requirements met at a 2.5% inclusion rate of residues, all three SB by-products appear best suited for gilts and gestating sows and different categories of horses. SBPS emerged as the promising protein source, as it supplied a strong amino acid profile. It also showed high concentrations of PUFAs and a more desirable n6:n3 ratio which offers numerous health benefits, especially for reproduction system. SBL, while also providing a solid amino acid profile, appeared to be a rich source of minerals, whereas the pomaces generally exhibited low mineral levels. SBPW showed the highest energy contribution based on ME predictions among the three by-products tested, suggesting it could serve as a valuable energy source for animals with higher energy requirements. On the other hand, SBPW’s weaker amino acid and mineral content may limit its overall nutritional value. All three residues were rich in vitamin E, while the pomaces were particularly high in β-carotene, both antioxidants, that can support reproduction, enhance immune function, and stabilize PUFAs, ensuring that the fatty acids remain intact and biologically active when included in animal feed. Consequently, supplementation with SB residues could be particularly beneficial for gestating or lactating animals and for those animals experiencing oxidative stress, such as during weaning, calving, growth, lactation, egg laying, racing, or heat stress. Additionally, previous studies have reported high concentrations of polyphenolic compounds in SB residues, especially in the leaves, which may further contribute to their antioxidant activity and improve the nutritional value of animal diets, thereby benefiting animal health (119, 120).

### 4.7 Limitations of the study

#### 4.7.1 Bioavailability and digestibility

Several limitations of the study should be noted. First of all, although this study provides preliminary results and highlights nutrients in which these materials are abundant or deficient, the bioavailability and absorption efficiency of these nutrients remain unknown. As a result, the reported percentages of daily requirement fulfillment are likely overestimated. Absorption efficiency differs among nutrients and animal species. Moreover, it is influenced by the nutrient’s chemical form, dietary antagonists, interactions within and across feed ingredients, as well as individual animal factors, making accurate predictions of absorption difficult. Digestibility experiments are therefore needed to assess how effectively animals can utilize the studied components. In addition, predicting the nutrient requirement fulfillment is challenging as animals can obtain minerals and vitamins from multiple on-farm sources (76).

Additional limitation of this study is that ME values presented in this study rely on prediction equations rather than direct digestibility determinations. Although these equations are widely used, they may not accurately reflect species-specific digestion, fermentation, or metabolic efficiency, particularly for unconventional feed ingredients such as SB residues. Consequently, the reported ME values should be interpreted as estimates, and future *in vivo* digestibility or gas-production trials will be essential to validate and refine these energy predictions.

In conclusion, feeding and digestibility trials would help evaluate the practical relevance of the materials, particularly in relation to animal health, environmental benefits (e.g., reduced methane and other emissions), and production efficiency.

#### 4.7.2 Antinutritional compounds and palatability

Another limitation of the study is that concentrations of potential antinutritional factors, such as phytic acid, tannins, or other compounds, were not measured. In addition, the palatability of the residues was not assessed, and it is possible that some materials may not be accepted by animals, potentially reducing voluntary feed intake regardless of their nutritional value. Future research should therefore evaluate both the presence of antinutritional factors and animal acceptance of residues to better understand their practical applicability in feed formulations.

#### 4.7.3 Storage and processing effects

Additionally, the obtained values do not account for the effects of on-farm storage on nutrient content. Extended storage may reduce nutrient concentration, potentially preventing daily requirements from being met and affecting growth and performance of animals. This concern applies not only to by-products but also to conventional feeds; however, if by-products are supplemented to provide specific, storage-sensitive nutrients, proper storage is essential for their preservation. Due to their large quantities, feed materials are less likely to be stored in the fridge and airtight packages that would reduce nutrient degradation.

Additional limitation, as mentioned before, is that vitamin analyses were based on a limited number of replicates; so obtained results should be interpreted as preliminary until validated with expanded replication.

#### 4.7.4 Comparability across feed evaluation systems

Lastly, in the process of choosing a feed evaluation system, we observed that nutrients daily requirements, especially mineral and vitamin requirements of animals, differ considerably depending on the feed evaluation system applied (34–36, 46–48, 52). Such differences likely come from differences in experimental data, underlying assumptions, safety margins, precision levels, animal genetics/breeds and their performance level as well as modeling approaches. As a result, the percentage of daily nutrient requirement fulfillment obtained in this study could vary if a different system were applied, and therefore, the results should be interpreted accordingly. It is important to note that the NRC generally reports lower nutrient requirements compared with other evaluation systems or studies, as previously highlighted for linoleic acid. This trend was also highlighted by Faccin et al. (121), who observed that swine nutritionists in general supplement vitamins and trace minerals well above the NRC (2012) requirement estimates (34). This further underscores the need to establish more precise and harmonized nutrient requirement guidelines across different feed evaluation systems.

## 5 Conclusion

This research provides a preliminary assessment of the potential of SB residues as feed ingredients. SBPS emerged as a promising protein source and PUFA-rich component, with possible benefits for animal health, reproduction, and growth. SBL showed good nutritional value through its favorable amino acid profile and high mineral content, supporting its use as a mineral-enhancing supplement. SBPW offered value primarily as an energy-dense ingredient. All residues were rich in vitamin E, and the pomaces contained notable amounts of β-carotene, suggesting antioxidant benefits for gestating animals, those experiencing oxidative stress, or animals with elevated metabolic demands. Furthermore, because these residues showed high ADIP content and consequently lower protein digestibility, feed formulations should account for processing methods and the addition of complementary protein sources to ensure nutritional balance. To fully unlock these by-products’ potential, future studies should focus on digestibility, nutrient bioavailability, and practical feeding trials under farm conditions. Moreover, further investigation into anti-nutritional factors and the impact of storage on the nutrient degradability of these residues is warranted. The incorporation of SB by-products into livestock diets could contribute to the valorization of agro-industrial residues and support more circular and sustainable feeding systems.

## Data Availability

The raw data supporting the conclusions of this article will be made available by the authors without undue reservation.
