# Supplementary material for: Nutrient assessment of sea buckthorn residues as potential feed ingredients
Source: Front Vet Sci. 2026 Feb 27;13:1767594. doi: 10.3389/fvets.2026.1767594 (PMC12982098; doi:10.3389/fvets.2026.1767594)
Supplement: Supplementary file 1 [file Table_1.docx]

Supplementary Material

**Table S1.** Linearity ranges and correlation coefficients of analyzed micro and macro elements.

| **Element** | **Linearity range (mg/L)** | **Correlation coefficient** |
| --- | --- | --- |
| Ca | 20 - 500 | 0.9998 |
| P | 32 - 400 | 0.9999 |
| Na | 4-50 | 0.9999 |
| K | 40 - 500 | 0.9999 |
| Mg | 8 - 200 | 0.9999 |
| Fe | 1-30 | 0.9996 |
| Cu | 0.5 - 10 | 0.9994 |
| Mn | 1-20 | 0.9999 |
| Zn | 1-30 | 0.9986 |
| Mo | 0.1 - 2 | 0.9992 |
| Ca | 20 - 500 | 0.9998 |

**Table S2.** Theoretical response correction factors for flame ionization detectors to convert to weight methyl ester

| **Fatty acid** | **Correction factor** | **References** |
| --- | --- | --- |
| 4:0 | 1.54 | **Christie, 1989 (1)** |
| 6:0 | 1.31 | **Christie, 1989 (1)** |
| 8:0 | 1.19 | **Christie, 1989 (1)** |
| 10:0 | 1.12 | **Christie, 1989 (1)** |
| 12:0 | 1.08 | **Christie, 1989 (1)** |
| 13:0 | 1.06 | **Christie, 1989 (1)** |
| 14:0 | 1.04 | **Christie, 1989 (1)** |
| 14:1 | 1.04 | **Christie, 1989 (1)** |
| 15:0 | 1.03 | **Christie, 1989 (1)** |
| 16:0 | 1.02 | **Christie, 1989 (1)** |
| 16:1 | 1.01 | **Christie, 1989 (1)** |
| 16:2 | 1.00 | **Christie, 1989 (1)** |
| 16:3 | 1.00 | **Christie, 1989 (1)** |
| 16:4 | 0.99 | **Christie, 1989 (1)** |
| 17:0 | 1.01 | **Christie, 1989 (1)** |
| 17:1 | 1.00 | **Christie, 1989 (1)** |
| 18:0 | 1.00 | **Christie, 1989 (1)** |
| 18:1 | 0.99 | **Christie, 1989 (1)** |
| 18:2 | 0.99 | **Christie, 1989 (1)** |
| 18:3 | 0.98 | **Christie, 1989 (1)** |
| 18:4 | 0.97 | **Christie, 1989 (1)** |

**Table S3.** Daily nutrient requirements for a 500 kg working horse with dry matter intake (DMI) of 2-2.5 % of body weight, a 550 kg pregnant mare with DMI of 2-2.5 % of body weight and a 500 kg lactating mare DMI of 2.5-3 % of body weight according to the Natural Resources Institute Finland (Luke, 2024) and, for selected nutrients, the National Research Council (NRC, 2007)

| **Items** | **Working horse, average workload** | **Pregnant mare** | **Lactating mare** | **Feed evaluation used** |
| --- | --- | --- | --- | --- |
| **Assumed** **DMI (kg)**  **:** | 12.5 | 12.4 | 12.5 | Luke (2) |
| **Weight (kg):** | 500 | 550 | 500 | Luke (2) |
| **Metabolizable Energy MJ/day** | 83.8 | 84 | 106 | Luke (2) |
| **Crude Protein, g** | 851 | 841 | 1530 | NRC (3) |
| **Digestible Crude Protein, g/ day** | 580 | 500 | 1000 | Luke (2) |
| **lysine g** | 36.5 | 36 | 84.4 | NRC (3) |
| **Ca, g** | 35 | 40 | 55 | Luke (2) |
| **P, g** | 23.5 | 29 | 38.5 | Luke (2) |
| **Mg, g** | 12.3 | 12 | 12.5 | Luke (2) |
| **Na, g** | 35 | 11 | 13 | NRC (3) |
| **K, g** | 40.8 | 26 | 48 | NRC (3) |
| **Fe, mg** | 550 | 900 | 900 | Luke (2) |
| **Cu, mg** | 92.5 | 125 | 125 | Luke (2) |
| **Mn, mg** | 450 | 400 | 500 | NRC (3) |
| **Zn, mg** | 550 | 400 | 400 | Luke (2) |
| **Vitamin A, mg^a^** | 13 | 11 | 11 | Luke (2) |
| **Vitamin D, mg^a^** | 0.1 | 0.2 | 0.2 | Luke (2) |
| **Vitamin E, mg^a^** | 1088 | 880 | 1100 | Luke (2) |
| **Thiamine, mg** | 46 | 30 | 38 | NRC (3) |
| **Riboflavin, mg** | 23 | 20 | 25 | NRC (3) |

^a^Vitamin A, D and E have been converted from IU to mg using Omni calculator (Omni Calculator, n.d.)

**Table S4.** Daily nutrient requirements for laying hens and broiler chicken according to the Natural Resources Institute Finland (Luke, 2024) and, for selected nutrients McDonald et al., 2011 and Estonian System (1995)

|  | **Laying hens** | **Broiler chicken** | **Feed Evaluation System Used** |
| --- | --- | --- | --- |
| **Assumed Feed Intake** | 120 g | 90g | Tikk & Piirsau, 1997 (4) |
| **Metabolizable Energy, MJ** | 1.3 | 1.1 | Luke^a^ (2) |
| **Crude Protein g** | 21.0 | 18.0 | Luke^a^ (2) |
| **Arginine, g^c^** | 1.1 | 0.9 | McDonald et al., 2011^b^ (5) |
| **Isoleucine, g^c^** | 0.9 | 0.6 | McDonald et al., 2011^b^ (5) |
| **Lysine, g^d^** | 0.9 | 0.9 | Luke^a^ (2) |
| **Methionine, g^d^** | 0.4 | 0.4 | Luke^a^ (2) |
| **Methionine + cystine, g^d^** | 0.8 | 0.6 | Luke^a^ (2) |
| **Threonine, g^d^** | 0.8 | 0.5 | Luke^a^ (2) |
| **Tryptophan, g^c^** | 0.2 | 0.1 | McDonald et al., 2011^b (^5) |
| **Valine, g^c^** | 1.0 | 0.7 | McDonald et al., 2011^b (^5) |
| **Linoleic acid, g** | 1.4 | 0.9 | Luke^a^ (2) |
| **Ca g** | 4.6 | 0.8 | Luke^a^ (2) |
| **P g** | 0.3 | 0.3 | Luke^a^ (2) |
| **Na g** | 0.2 | 0.1 | Luke^a^ (2) |
| **K g** | 0.4 | 0.4 | Luke^a^ (2) |
| **Mg mg** | 66.0 | 54.0 | Luke^a^ (2) |
| **Fe mg** | 9.0 | 8.1 | Luke^a^ (2) |
| **Cu mg** | 0.9 | 0.8 | Luke^a^ (2) |
| **Mn mg** | 8.4 | 5.9 | Luke^a^ (2) |
| **Zn mg** | 7.2 | 5.0 | Luke^a^ (2) |
| **Vitamin A, mg^e^** | 0.3 | 0.2 | Luke^a^ (2) |
| **Vitamin D, mg^e^** | 0.01 | 0.003 | Luke^a^ (2) |
| **Vitamin E, mg^e^** | 1.8 | 1.8 | Luke^a^ (2) |
| **Thiamine, vitamin B1, mg** | 0.2 | 0.2 | Luke^a^ (2) |
| **Riboflavin, vitamin B2, mg** | 0.5 | 0.4 | Luke^a^ (2) |
| **Pyridoxine, vitamin B6, mg** | 0.4 | 0.3 | Luke^a^ (2) |
| **Pantothenic acid, vitamin B5, mg** | 1.0 | 1.0 | Luke^a^ (2) |
| **Niacin, vitamin B3, mg** | 4.2 | 0.0 | Luke^a^ (2) |

^a^Values from Luke were reported as g, mg, or IU/kg. To express them on a daily basis (g, mg, or IU/day), they were multiplied by the assumed feed intake suggested by Tikk & Piirsalu (1997).

^b^values reported by McDonald et al. (2017) in g, mg, or IU/kg were converted to daily values (g, mg, or IU/day) using the assumed feed intake from Tikk & Piirsalu (1997).

^c^ Amino acid requirements for laying hens reported by McDonald et al. (2011) are expressed as standardized ileal digestible (SID) values. and for broiler chicken reported by McDonald et al., (2011) are expressed as total tract digestible (SID) values

^d^ In Luke it is not specified whether reported values refer to standardized ileal digestible (SID) values or total amino acid requirement values

^e^ Vitamin A, D and E have been converted from IU to mg using Omni calculator (Omni Calculator, n.d.)

**Table S5.** Daily nutrient requirements for weaned piglets, growing pigs, gilts, pregnant sows and lactating sows according to the Danish nutrient standard (2020) and, for selected nutrients, the National Research Council (NRC, 2012) and Estonian system (1995)

|  | **Weaned piglet** | **Finishers** | **Finishers** | **Gilts** | **Pregnant sow** | **Pregnant sow** | **Lactating sow** | **Feed evaluation system used** |
| --- | --- | --- | --- | --- | --- | --- | --- | --- |
| **Weight for growing stages/ gestation** | 9-15 kg, 1.65-1.8 FUgp / kg gain, | 30-60 kg, 2.45–2.6 FUgp / kg gain |  | 60-110 kg | early gestation, 0-110 days | late gestation, 110-117 days |  | **Danish^a^ (6)** |
|  | 7-11kg | 25-50kg; 50-75kg | 75-100; 100-135 kg | 50-75; 75-100kg | <90 days | >90 days |  | **NRC (7)** |
| **Assumed feed intake (kg):** | 0.5 | 1.9 | 2.8 | 2.525 | 2.2 | 2.6 | 6.3 | **NRC (7)** |
| **Metabolizable energy, MJ** | 9.7 | 25.0 | 29.2 | 30.5 | 28.2 | 33.4 | 82.4 | **NRC (7)** |
| **Crude protein, g** | 180^h^ | 314^h^ | 399^h^ | 348^h^ | 283.5^h^ | 338^h^ | 855^h^ | **Estonian (8)** |
| **Histidine, g** | 2.5 | 6.6^i^ | 7.2^j^ | 7.2^k^ | 3.3^l^ | 5.0^m^ | 21.9^n^ | **NRC (7)** |
| **Isoleucine, g** | 3.7 | 9.95^i^ | 11.1^j^ | 11.1^k^ | 5.6^l^ | 8.2^m^ | 30.7^n^ | **NRC (7)** |
| **Leucine, g** | 7.2 | 18.9^i^ | 20.6^j^ | 20.9^k^ | 8.6^l^ | 14.7^m^ | 61.5^n^ | **NRC (7)** |
| **Lysine, g** | 7.2 | 18.8^i^ | 20.4^j^ | 20.7^k^ | 9.5^l^ | 15.5^m^ | 54.5^n^ | **NRC (7)** |
| **Methionine, g** | 2.1 | 5.5^i^ | 6.0^j^ | 6.0^k^ | 2.7^l^ | 4.3^m^ | 14.7^n^ | **NRC (7)** |
| **Methionine + cysteine, g** | 4.1 | 11.0^i^ | 12.3^j^ | 12.2^k^ | 6.7^l^ | 10.9^m^ | 30.1^n^ | **NRC (7)** |
| **Phenylalanine, g** | 4.3 | 11.4^i^ | 12.5^j^ | 12.5^k^ | 5.4^l^ | 8.8^m^ | 30.0^n^ | **NRC (7)** |
| **Phenylalanine + tyrosine, g** | 6.8 | 18.1^i^ | 20.0^j^ | 20.0^k^ | 9.6^l^ | 15.5^m^ | 62.6^n^ | **NRC (7)** |
| **Threonine, g** | 4.4 | 12.1^i^ | 13.9^j^ | 13.6^k^ | 7.8^l^ | 12.0^m^ | 36.6^n^ | **NRC (7)** |
| **Tryptophan, g** | 1.2 | 3.2^i^ | 3.6^j^ | 3.6^k^ | 1.8^l^ | 3.1^m^ | 10.3^n^ | **NRC (7)** |
| **Valine, g** | 4.7 | 12.6^i^ | 14.0^j^ | 14.0^k^ | 7.2 | 11.6 ^m^ | 47.3^n^ | **NRC (7)** |
| **Linoleic acid, g^d^** | 0.7 | 1.8 | 2.7 | / | 2.1 | 2.1 | 6.0 | **NRC (7)** |
| **Ca, g^c^** | 4.0 | 13.2 | 17.5 | 17.5 | 13.5 | 16 | 48.1 | **Danish^a^ (6)** |
| **P, g^d^** | 4.2 | 9.7 | 11.9 | 11.3 | 8.6 | 13.6 | 36.1 | **NRC (7)** |
| **Na, g** | 1.2 | 3.3 | 4.1 | 4.0 | 3.3 | 3.9 | 10.0 | **Danish^a^ (6)** |
| **K, g** | 1.4 | 4.9 | 7.3 | 6.6 | 5.4 | 6.4 | 16.7 | **Danish^a^ (6)** |
| **Mg, mg** | 0.5 | 1.6 | 1.8 | 1.9 | 1.3 | 1.5 | 6.7 | **Danish^a^ (6)** |
| **Fe, mg^e^** | 84.0 | 155.4 | 233.8 | 212.1 | 174.2 | 205.9 | 534.2 | **Danish^a^ (6)** |
| **Cu, mg** | 3.4 | 11.6 | 17.5 | 15.9 | 13.1 | 15.4 | 40.1 | **Danish^a^ (6)** |
| **Mn, mg** | 22.4 | 77.7 | 116.9 | 106.1 | 87.1 | 103.0 | 267.1 | **Danish^a^ (6)** |
| **Zn, mg^f^** | 56.0 | 194.3 | 292.3 | 265.1 | 217.8 | 257.4 | 667.8 | **Danish^a^ (6)** |
| **Vitamin A, mg^g^** | 0.8 | 2.3 | 3.5 | 3.2 | 5.2 | 6.2 | 16.0 | **Danish^a^ (6)** |
| **Vitamin D, mg^g^** | 0.0 | 0.0 | 0.0 | 0.0 | 0.0 | 0.1 | 0.1 | **Danish^a^ (6)** |
| **Vitamin E, mg^g^** | 52.6 | 52.2 | 78.5 | 71.2 | 58.5 | 69.1 | 739.5 | **Danish^a^ (6)** |
| **Thiamine, vitamin B1, mg** | 1.1 | 3.9 | 5.8 | 5.3 | 4.4 | 5.1 | 13.4 | **Danish^a^ (6)** |
| **Riboflavin, vitamin B2, mg** | 2.2 | 3.9 | 5.8 | 5.3 | 10.9 | 12.9 | 33.4 | **Danish^a^ (6)** |
| **Niacin, vitamin B3, mg** | 11.2 | 38.9 | 58.5 | 53.0 | 43.6 | 51.5 | 133.6 | **Danish^a^ (6)** |
| **Pantothenic acid, vitamin B5, mg** | 5.6 | 19.4 | 29.2 | 26.5 | 32.7 | 38.6 | 100.2 | **Danish^a^ (6)** |
| **Pyridoxine, vitamin 6, mg** | 1.7 | 5.8 | 8.8 | 8.0 | 6.5 | 7.7 | 20.0 | **Danish^a^ (6)** |

^a^ Values from the Danish nutrient standards (Tybrik et al., 2020). Values are originally expressed as g, mg, or IU/FU. To convert these to g, mg, or IU/kg, the values were multiplied by the following factors based on the energy content of conventional complete diets: weaned piglets ×1.12; growing pigs and gilts ×1.05; gestating sows ×0.99; lactating sows ×1.06.

^b^ Linoleic acid requirement values were obtained from the NRC (2012). For each pig category, values were averaged across the relevant weight ranges: weaned piglets (7–11 kg and 11–25 kg), growing pigs (25–50 kg and 50–75 kg; 75–100 kg and 100–135 kg), and gilts (50–75 kg and 75–100 kg). For gilts over 120 kg, as well as for pregnant and lactating sows, a single value was used.

^c^ Calcium requirements, when 150-250% phytase is supplemented

^d^ Phosphorus requirement values were obtained from the NRC (2012). For each pig category, values were averaged across the relevant weight ranges: weaned piglets (7–11 kg and 11–25 kg), growing pigs (25–50 kg and 50–75 kg), finishers (75–100 kg and 100–135 kg), and gilts (50–75 kg and 75–100 kg). For gilts over 120 kg, pregnant and lactating sows, a single value was used.

^e^ For the iron requirement, at least 100 mg should be provided as an easily soluble iron salt.

^f^ Zinc requirements when 0-150% phytase is supplemented

^g^Vitamin A, D and E have been converted from IU to mg using Omni calculator (Omni Calculator, n.d.)

^h^Protein requirement values represent the crude protein daily requirements according to the Estonian feeding system (Oll & Tölp, 1995).

^i^ Values represent the total amino acid requirements for growing pigs, calculated as the average of NRC-recommended requirements (2012) for the 25–50 kg and 50–75 kg weight ranges.

^j^Values represent the total amino acid requirements, calculated as the average of NRC-recommended values (2012) for growing pigs weighing 75–100 kg and 100–135 kg

^k^ Values represent the total amino acid requirements, calculated as the average of NRC-recommended values (2012) for gilts weighing 50–75 kg and 75–100 kg.

^l^ Values represent the total amino acid requirements, averaged from NRC recommendations (2012) for pregnant sows (140–205 kg BW, <90 days of gestation, 1st–4th parity), with an anticipated weight gain of 45–65 kg and an expected litter size of 12.5–15.5 piglets.

^m^ Values represent the total amino acid requirements, averaged from NRC recommendations (2012) for pregnant sows (140–205 kg BW, >90 days of gestation, 1st–4th parity), with an anticipated weight gain of 45–65 kg and an expected litter size of 12.5–15.5 piglets.

^n^ Values represent NRC amino acid requirements (2012) averaged for lactating sows (175–210 kg post-farrowing BW, parity 1 or 2+, 11–11.5 litter size, 21-d lactation, 190–270 g daily gain

**Table S6.** Daily nutrient requirements for heifers (200 and 400kg), dairy cows (650 kg, ECM 35 kg), dry cows and calves according to the Natural Resources Institute Finland (Luke, 2024) the National Research Council (NRC, 2021) and for selected nutrients NorFor (2011) and Estonian System (1995, 2019)

|  | **Heifers, 200kg** | **Heifers, 400 kg** | **Feed evaluation system used** | **Dairy cows, 650 kg, ECM 35 kg** | **Feed evaluation system used** | **Dry cows** | **Feed evaluation system used** | **Calves** | **Feed evaluation system used** |
| --- | --- | --- | --- | --- | --- | --- | --- | --- | --- |
| **DMI, kg^c^** | 4.6 | 7.1 | **NorFor (9)** | 24.7^j^ | **Estonian (8)** | 12.3 | **NRC^a^(10)** | 2^t^ | **Estonian^b^ (12)** |
| **ME, MJ** | 62.5^e^ | 98^f^ | **Luke (2)** | 242.7^k^ | **Luke (2)** | 115.5^p^ | **NRC^a^(10)** | 26^u^ | **NRC (10)** |
| **CP, g** | 658 | 867 | **NRC^c^ (10)** | 4199^l^ | **Penn state (11)** | 1758.9^q^ | **NRC^a^(10)** | 357.8^u^ | **NRC (10)** |
| **Ca, g** | 30^g^ | 37^g^ | **Luke (2)** | 122.5^m^ | **Luke (2)** | 47.97^q^ | **NRC^a^(10)** | 15^v^ | **NRC (10)** |
| **P, g** | 17^g^ | 20^g^ | **Luke (2)** | 77.5^m^ | **Luke (2)** | 25.8^q^ | **NRC^a^(10)** | 7.4^v^ | **NRC (10)** |
| **Na, g** | 5^g^ | 9^g^ | **Luke (2)** | 36.5^m^ | **Luke (2)** | 20.9^q^ | **NRC^a^(10)** | 4.4^v^ | **NRC (10)** |
| **K, g** | 27.4^h^ | 42.8^h^ | **NorFor (9)** | 128^m^ | **Luke (2)** | 84.9^q^ | **NRC^a^(10)** | 12^v^ | **NRC (10)** |
| **Mg (inside), g** | 6 ^g^ | 10 ^g^ | **Luke (2)** | 34^m^ | **Luke (2)** | 17.2^q^ | **NRC^a^(10)** | 3^v^ | **NRC (10)** |
| **Fe, mg** | 457^i^ | 713^i^ | **Luke (2)** | 2470^n^ | **Luke (2)** | 185^s^ | **NRC^a^(10)** | 120^v^ | **NRC (10)** |
| **Cu, mg** | 46 ^i^ | 71^i^ | **Luke (2)** | 247^n^ | **Luke (2)** | 234^s^ | **NRC^a^(10)** | 24^v^ | **NRC (10)** |
| **Mn, mg** | 228^i^ | 357^i^ | **Luke (2)** | 988^o^ | **Luke (2)** | 529^s^ | **NRC^a^(10)** | 80^v^ | **NRC (10)** |
| **Zn, mg** | 183^i^ | 285^i^ | **Luke (2)** | 1235^n^ | **Luke (2)** | 381^s^ | **NRC^a^(10)** | 110^v^ | **NRC (10)** |
| **Vitamin A, mg^d^** | 3 | 5 | **Luke (2)** | 24 | **Luke (2)** | 25^s^ | **NRC^a^(10)** | 2^w^ | **NRC (10)** |
| **Vitamin D mg^d^** | 0.03 | 0.05 | **Luke (2)** | 1 | **Luke (2)** | 0.6^s^ | **NRC^a^(10)** | 0.1^w^ | **NRC (10)** |
| **Vitamin E, mg^d^** | 77 | 119 | **Luke (2)** | 249 | **Luke (2)** | 1494^r^ | **NRC^a^(10)** | 90^w^ | **NRC (10)** |
| **Lysine, g** |  |  |  |  |  |  | **NRC^a^(10)** | 19^x^ | **Estonian^b^ (12)** |
| **Methionine g** |  |  |  |  |  |  |  | 9^x^ | **Estonian^b^(12)** |

^a^ NRC requirements apply for dry cow, >21 days prepartum, weighing 740kg

^b^Estonian recommendations for calves were taken from MTÜ Piimaklaster, *Uuendnoorkarja söötmisstrateegia vabapidamisega farmides* (2019), and refer to starter feed requirements. Originally expressed as % or mg/IU per kg DM, the values were converted to daily requirements by multiplying by the assumed dry matter intake (DMI) and by additional 10 if they were expressed as %

**^c^** NorFor equation was used for determining dry matter intake of heifers (200 and 400 kg) and it has been based on weight of the animal

dVitamin A, D and E have been converted from IU to mg using Omni calculator (Omni Calculator, n.d.)

^e^ME requirements refer to heifers weighing 150–250 kg with a growth rate of 1.0 kg/day. Values for the heifers weighing 150–200 kg and 200–250 kg were averaged,

^f^ ME requirements refer to heifers weighing 350–450 kg with a growth rate of 1.0 kg/day. Values for the heifers weighing 350–400 kg and 400–450 kg were averaged

^g^ macroelement requirements refer to heifers with a growth rate of 1.0 kg/day.

^h^ K requirements were calculated based on NorFor equation

^i^ microelement requirements are specified for young cattle. Originally expressed as g, mg, or IU/kg DM, they were converted to daily requirements by multiplying the values by the assumed DMI derived from NorFor equation

**^j^** Estonian system was used for determining dry matter intake of dairy cow and it has been calculated based on weight of the animal: for cows in mid lactation it is 3.8 % of animals body weight

^k^ME requirement has been calculated as 5.15 MJ/kg ECM × ECM, kg/day

^l^Protein requirements for dairy cows have been taken from Penn State University recommendation, initially expressed as % DM they have been converted to g/kg DM and then multiplied by assumed DMI derived from Estonian system

^m^Macroelement requirements were calculated as the average of values for a 650 kg cow producing 30 kg and 40 kg of milk.

Protein requirements have been taken from Penn State University recommendation, initially expressed as % DM they have been converted to g/kg DM and then multiplied by assumed DMI derived from Estonian system

^n^Microelement requirements, originally expressed as g, mg, or IU/kg DM, were converted to daily requirements by multiplying them with the assumed DMI, as calculated according to the Estonian system.

^o^ Luke specifies that during the first 3 months of lactation Mn requirements can be 80 mg/kg DM

^p^ Metabolizable energy (ME) was originally expressed as Mcal/day and was converted to MJ/day using a factor of 4.184.

^r^ values were originally expressed as % of DM. They were converted to g / kg DM and then multiplied by suggested DMI

^s^ The values, originally expressed as mg or IU/kg DM, were converted to a daily basis by multiplying them with the assumed DMI.

^t^ DMI refers to 2-month-old calves

^u^ These values represent the average daily energy and protein requirements of calves weighing 75–85 kg, fed exclusively solid feeds, with average daily gains between 400 and 800 g/day. Metabolizable energy (ME) was originally expressed as Mcal/day and was converted to MJ/day using a factor of 4.184.

^v^NRC-recommended mineral concentrations in starter feeds (DM basis) for calves weighing 35–125 kg and growing at 0.5–1.2 kg/day were originally expressed as % of DM (for macroelements) and mg/kg DM (for microelements). Microelement concentrations were first converted to g/kg DM and then all mineral values were multiplied by the suggested DMI, as obtained from the Estonian feeding system, to calculate daily intake

^w^ Vitamin A, D and E have been converted from IU to mg using Omni calculator (Omni Calculator, n.d.). Vitamin recommendation values assume an 80-kg calf consuming 2.4 kg of starter DM. They have initially been expressed as IU/kg DM and have been converted to IU/ day by being multiplied by assumed DMI, obtained from Estonian feeding system

^x^ initially presented as g/ kg DM, values were multiplied by assumed DMI to be converted to daily requirements

**Table S7.** Proximate composition in sea buckthorn leaves (SBL), sea bucktorn pomace with seeds (SBPS) and sea buckthorn pomace without seeds (SBPW) (expressed as % DM) and conventional feed ingredients

| Reference | **Original research** | | | **INRA (13)** | | | | | | | | | | | | | **NorFor (14)** | | | | | | | |
| --- | --- | --- | --- | --- | --- | --- | --- | --- | --- | --- | --- | --- | --- | --- | --- | --- | --- | --- | --- | --- | --- | --- | --- | --- |
|  | **SBL** | **SBPS** | **SBPW** | **Barley^a^** | **Maize^a^** | **Oats^a^** | **Rye^a^** | **Triticale^a^** | **Wheat durum^a^** | **Wheat soft^a^** | **Rapeseed Meal, oil <5 %^a^** | **Soybean meal, oil <5, 50 % protein + oil ^a^** | **Peas^a^** | **sunflower meal, oil <5 %, dehulled^a^** | **faba beans, coloured flowers^a^** | **linseed whole^a^** | **linseed meal, oil < 5% ^a^** | **white clover^b,c^** | **red clover^b,d^** | **silage grass clover^b, e^** | **clover grass hay, high OMD ^b,f^** | **straw, spring barley^b,g^** | **Italian ryegrass, spring, early^b,h^** | **Grass, mixed meadow. Medium dig.^b,1^** |
| **Gross Energy** | 18.4 | 22.5 | 22.7 | 18.3 | 18.6 | 19.5 | 18 | 18.1 | 18.5 | 18.2 | 19.3 | 19.7 | 18.3 | 19.4 | 18.7 | 27 | 19.5 | 6.05 | 5.72 | 6.06 | 5.6 | 3.08 | 7.01 | 6.22 |
| **ME (pigs)** | 13.6 | 15.1 | 15.6 | 14.3 / 14.5 | 15.9 / 16.5 | 12.3 / 12.9 | 15 / 15.2 | 15 / 15.2 | 15.3 / 15.6 | 15.4 / 15.6 | 12.2 / 12.9 | 15.8 / 16.5 | 15.3 / 15.7 | 9.1 / 10.3 | 14.7 / 15 | 20.1 / 20.6 | 13.8 / 14.2 | n/a | n/a | n/a | n/a | n/a | n/a | n/a |
| **ME (broiler)** | 10.1 | 15.4 | 17.0 | 12.5 | 15 | 10.7 | 11.3 | 13.5 | 13.7 | 13.8 | 6.8 | 11.2 | 11.7 | 6.2 | 10.9 | 17.1 | 9.8 | n/a | n/a | n/a | n/a | n/a | n/a | n/a |
| **ME (horses)** | 10.9 | 13.0 | 15.6 | n/a | n/a | n/a | n/a | n/a | n/a | n/a | n/a | n/a | n/a | n/a | n/a | n/a | n/a | n/a | n/a | n/a | n/a | n/a | n/a | n/a |
| **ME (ruminants)** | 8.1 | 9.7 | 13.7 | 12.2 | 13.6 | 11.5 | 12.9 | 12.9 | 13.1 | 13 | 11.4 | 14.1 | 13.5 | 7.9 | 13.7 | 18.7 | 11.8 | n/a | n/a | n/a | n/a | n/a | n/a | n/a |
| **CP** | 13.6 | 22.5 | 9.6 | 11.3 | 8.8 | 10.8 | 9.8 | 11.5 | 16.4 | 12.6 | 38.1 | 55.2 | 23.3 | 30.7 | 29.5 | 22.5 | 36.4 | 26.8 | 22.5 | 14.9 | 13.2 | 5.1 | 25 | 14.3 |
| **EE** | 2.9 | 15.2 | 20.1 | 1.9 | 4.1 | 5.4 | 1.4 | 1.4 | 2 | 1.6 | 2.4 | 1.7 | 1.4 | 2.1 | 1.5 | 37.6 | 3.9 | 3.6 | 3.2 | 3.3 | 2.4 | 1.3 | 4.9 | 3.9 |
| **CF** | 14.3 | 18.9 | 15.4 | 5.4 | 2.6 | 13.1 | 2.3 | 2.9 | 3 | 2.7 | 14.3 | 4.4 | 6.4 | 29.5 | 9 | 9.9 | 11.3 | n/a | n/a | n/a | n/a | n/a | n/a | n/a |
| **NDF** | 24.7 | 35.8 | 32.6 | 21.5 | 12.4 | 35.7 | 14.8 | 15 | 15.9 | 14.7 | 31.6 | 10.5 | 14.6 | 46.8 | 15.5 | 25.9 | 24.8 | 26.2 | 33.5 | 48.3 | 42.2 | 77.1 | 35 | 57.2 |
| **ADF** | 21.0 | 29.1 | 26.7 | 6.5 | 3.1 | 16.3 | 3.3 | 3.8 | 4.2 | 3.8 | 20.7 | 5.7 | 7.3 | 33.6 | 11.2 | 14.1 | 15.6 | n/a | n/a | n/a | n/a | n/a | n/a | n/a |
| **Starch** | 0.7 | 0.3 | 0.2 | 59.9 | 73.9 | 42.1 | 62 | 67.8 | 63.6 | 69.1 | 6.3 | 6.3 | 51.3 | 3.7 | 44.2 | 6 | 9.4 | 2 | 1 | 1 | 1.5 | 0 | 0 | 0 |
| **EE** | 2.9 | 15.2 | 20.1 | 1.9 | 4.1 | 5.4 | 1.4 | 1.4 | 2 | 1.6 | 2.4 | 1.7 | 1.4 | 2.1 | 1.5 | 37.6 | 3.9 | 3.6 | 3.2 | 3.3 | 2.4 | 1.3 | 4.9 | 3.9 |
| **Ash** | 5.8 | 2.1 | 2.0 | 2.5 | 1.4 | 2.9 | 2 | 2 | 2.1 | 1.8 | 7.6 | 7.3 | 3.5 | 6.8 | 3.8 | 4.2 | 6.5 | 11.2 | 16.7 | 7.9 | 8.6 | 6 | 1.1 | 7.7 |

^1^ Values have been taken from INRA feed tables

^2^ Values have been taken from NorFor feed tables

^3^ White clover from the NorFor tables (code 006-0093) was used

^4^ Red clover from the NorFor tables (code 006-0093) was used

^5^ Silage grass clover from the NorFor tables (code 006-0165) was used

^6^ Clover grass hay, with high Organic Mater Digestbility (OMD) from the NorFor tables (code 006-0403) was used

^7^ Straw, spring barley from the NorFor tables (code 006-0386) was used

^8^ Italian ryegrass, spring, early from the NorFor tables (code 006-0084) was used

^9^ Grass, mixed meadow with medium digestibility (dig) from the NorFor tables (code 006-0503) was used

**Table S8.** ADIP in sea buckthorn leaves (SBL), sea bucktorn pomace with seeds (SBPS) and sea buckthorn pomace without seeds (SBPW) and conventional feed ingredients, expressed as g/kg DM

|  | **ADIP, g/kg** | Reference |
| --- | --- | --- |
| **SBL** | 25.8 |  |
| **SBPS** | 15.7 |  |
| **SBPW** | 11.8 |  |
| **Grass, young** | 1.3 | **McDonald et al., 2011 (5)** |
| **Grass, mature** | 0.7 | **McDonald et al., 2011 (5)** |
| **Kale** | 2.3 | **McDonald et al., 2011 (5)** |
| **Barley, whole crop** | 2.2 | **McDonald et al., 2011 (5)** |
| **Grass silage, young** | 1.3 | **McDonald et al., 2011 (5)** |
| **Grass silage, mature** | 0.5 | **McDonald et al., 2011 (5)** |
| **Lucerne** | 1.7 | **McDonald et al., 2011 (5)** |
| **Maize, whole crop** | 2.7 | **McDonald et al., 2011 (5)** |
| **Red clover** | 2.2 | **McDonald et al., 2011 (5)** |
| **Grass hay, poor quality** | 1.2 | **McDonald et al., 2011 (5)** |
| **Grass hay, good quality** | 0.5 | **McDonald et al., 2011 (5)** |
| **Lucerne, early-flowering** | 2.1 | **McDonald et al., 2011 (5)** |
| **Barley straw** | 1 | **McDonald et al., 2011 (5)** |
| **Oat straw** | 0.6 | **McDonald et al., 2011 (5)** |
| **Wheat straw** | 0.8 | **McDonald et al., 2011 (5)** |
| **Fodder beat** | 0.9 | **McDonald et al., 2011 (5)** |
| **Potatoes** | 1.6 | **McDonald et al., 2011 (5)** |
| **Sugar beet pulp, dried** | 1.6 | **McDonald et al., 2011 (5)** |
| **Sugar beet pulp, dried mollased** | 0.9 | **McDonald et al., 2011 (5)** |
| **Swedes** | 0.2 | **McDonald et al., 2011 (5)** |
| **Sweet potatoes** | 0.2 | **McDonald et al., 2011 (5)** |
| **Turnips** | 1.6 | **McDonald et al., 2011 (5)** |
| **Barley** | 0.4 | **McDonald et al., 2011 (5)** |
| **Barley, brewers grain** | 3.9 | **McDonald et al., 2011 (5)** |
| **Grain, distillers grains** | 15.8 | **McDonald et al., 2011 (5)** |
| **Grain, distillers, dark grains** | 10 | **McDonald et al., 2011 (5)** |
| **Maize** | 1.3 | **McDonald et al., 2011 (5)** |
| **Maize, gluten feed** | 1.4 | **McDonald et al., 2011 (5)** |
| **Maize glutean, meal** | 6.4 | **McDonald et al., 2011 (5)** |
| **Oats** | 0.4 | **McDonald et al., 2011 (5)** |
| **Oats, naked** | 0.5 | **McDonald et al., 2011 (5)** |
| **Rice bran, extracted** | 1.4 | **McDonald et al., 2011 (5)** |
| **Wheat** | 0.4 | **McDonald et al., 2011 (5)** |
| **Wheat feed** | 0.4 | **McDonald et al., 2011 (5)** |
| **Coconut meal** | 2.5 | **McDonald et al., 2011 (5)** |
| **Cotton seed meal, undecorticated** | 3.2 | **McDonald et al., 2011 (5)** |
| **Cotton seed meal, decorticated** | 2 | **McDonald et al., 2011 (5)** |
| **Groundnut meal, decorticated** | 2 | **McDonald et al., 2011 (5)** |
| **Linseed meal** | 2 | **McDonald et al., 2011 (5)** |
| **Palm kernel meal** | 3 | **McDonald et al., 2011 (5)** |
| **Rapeseed meal** | 3.6 | **McDonald et al., 2011 (5)** |
| **Soyabean meal** | 2.2 | **McDonald et al., 2011 (5)** |
| **Sunflower meal, undecorticated** | 2.5 | **McDonald et al., 2011 (5)** |
| **Sunflower meal, decorticated** | 2 | **McDonald et al., 2011 (5)** |
| **Beans, field** | 0.5 | **McDonald et al., 2011 (5)** |
| **Peas** | 4.8 | **McDonald et al., 2011 (5)** |

**Table S9.** Concentration of minerals in sea buckthorn leaves (SBL), sea bucktorn pomace with seeds (SBPS) and sea buckthorn pomace without seeds (SBPW) and conventional feed ingredients, expressed as g/kg DM (microelements) and mg/kg DM (microelements)

| **References** | **Original research** | | | **INRA (13)** | | | | | | | | | | | | | | **NorFor (14)** | | | | | | |
| --- | --- | --- | --- | --- | --- | --- | --- | --- | --- | --- | --- | --- | --- | --- | --- | --- | --- | --- | --- | --- | --- | --- | --- | --- |
|  | **SBL** | **SBPS** | **SBPW** | **Barley^a^** | **Maize^a^** | **Oats^a^** | **Rye^a^** | **Triticale^a^** | **Wheat durum^a^** | **Wheat soft^a^** | **Rapeseed Meal, oil <5 %^a^** | **Soybean meal, oil <5, 50 % protein + oil ^a^** | **Peas^a^** | **sunflower meal, oil <5 %, dehulled^a^** | **faba beans, coloured flowers^a^** | **linseed whole^a^** | **linseed meal, oil < 5% ^a^** | **white clover^b,c^** | **red clover^b,d^** | **silage grass clover^b, e^** | **clover grass hay, high OMD ^b,f^** | **straw, spring barley^b,g^** | **Italian ryegrass, spring, early^b,h^** | **Grass, mixed meadow. Medium dig.^b,1^** |
| **Ca^j^** | 16.6 | 0.9 | 1.2 | 0.8 | 0.5 | 1.1 | 0.7 | 0.7 | 0.8 | 0.7 | 8.6 | 3.9 | 1.2 | 4.3 | 1.5 | 3.0 | 4.5 | 14.7 | 13.3 | 5.5 | 5.4 | 4.6 | 6.3 | 4.1 |
| **P^j^** | 0.8 | 3.5 | 1.2 | 3.9 | 2.9 | 3.6 | 3.5 | 3.8 | 3.9 | 3.6 | 12.7 | 7.1 | 4.4 | 11.0 | 5.5 | 6.3 | 10.0 | 3.1 | 3.0 | 2.7 | 2.9 | 0.9 | 4.3 | 2.8 |
| **Na^j^** | 0.8 | 0.2 | 0.2 | 0.1 | 0.0 | 0.1 | 0.0 | 0.1 | 0.1 | 0.1 | 0.3 | 0.1 | 0.0 | 0.1 | 0.1 | 0.5 | 1.1 | 2.9 | 0.9 | 0.7 | 1.4 | 1.4 | 2.3 | 0.3 |
| **K^j^** | 5.5 | 8.2 | 7.8 | 5.5 | 3.6 | 5.1 | 5.2 | 5.7 | 4.9 | 4.5 | 14.1 | 24.3 | 11.4 | 15.9 | 11.6 | 8.2 | 11.3 | 18.1 | 19.1 | 21.7 | 24.3 | 20.0 | 35.0 | 22.0 |
| **Mg^j^** | 2.5 | 0.8 | 0.3 | 1.3 | 1.2 | 1.1 | 1.2 | 1.2 | 1.2 | 1.1 | 4.6 | 3.2 | 1.3 | 5.3 | 1.9 | 4.0 | 5.2 | 2.1 | 2.9 | 1.9 | 1.4 | 0.9 | 1.9 | 1.4 |
| **Fe^k^** | 143.3 | 31.1 | 17.0 | 25.0 | 53.0 | 6.0 | 65.0 | 46.0 | 56.0 | 56.0 | 183.0 | 201.0 | 108.0 | 198.0 | 68.0 | 164.0 | 164.0 | 150.0 | 130.0 | 151.0 | 80.0 | 470.0 | 230.0 | 220.0 |
| **Cu^k^** | 4.4 | 8.4 | 6.2 | 9.0 | 3.0 | 4.0 | 6.0 | 6.0 | 5.0 | 5.0 | 9.0 | 17.0 | 8.0 | 34.0 | 14.0 | 13.0 | 18.0 | 9.0 | 7.0 | 6.3 | 5.6 | 3.0 | 11.0 | 5.0 |
| **Mn^k^** | 93.7 | 13.5 | 11.0 | 18.0 | 10.0 | 47.0 | 53.0 | 24.0 | 35.0 | 35.0 | 68.0 | 44.0 | 15.0 | 39.0 | 8.0 | 32.0 | 39.0 | 60.0 | 60.0 | 58.0 | 56.0 | 30.0 | 50.0 | 70.0 |
| **Zn^k^** | 13.5 | 18.0 | 7.1 | 34.0 | 24.0 | 27.0 | 25.0 | 28.0 | 30.0 | 30.0 | 78.0 | 57.0 | 39.0 | 86.0 | 36.0 | 50.0 | 66.0 | 30.0 | 29.0 | 29.0 | 21.0 | 147.0 | 50.0 | 57.0 |
| **Mo^k^** | 0.1 | 0.2 | 0.2 | 0.5 | 0.5 | 0.9 | 0.6 | 0.6 | 0.5 | 0.5 | 2.0 | 3.0 | 2.0 | 0.7 | 0.7 | 0.2 | 1.0 | n/a | n/a | 1.4 | 0.7 | 0.2 | n/a | n/a |

**^a^** Values have been taken from INRA feed tables

^b^ Values have been taken from NorFor feed tables

^c^ White clover from the NorFor tables (code 006-0093) was used

^d^ Red clover from the NorFor tables (code 006-0093) was used

^e^ Silage grass clover from the NorFor tables (code 006-0165) was used

^f^ Clover grass hay, with high Organic Mater Digestbility (OMD) from the NorFor tables (code 006-0403) was used

^g^ Straw, spring barley from the NorFor tables (code 006-0386) was used

^h^ Italian ryegrass, spring, early from the NorFor tables (code 006-0084) was used

^i^ Grass, mixed meadow with medium digestibility (dig) from the NorFor tables (code 006-0503) was used

^j^ expressed as g/ kg DM

^k^ expressed as mg//kg DM

**Table S10.** Amino acid concentration of sea buckthorn leaves (SBL), sea bucktorn pomace with seeds (SBPS) and sea buckthorn pomace without seeds and conventional feed ingredients, expressed as g/kg DM

| **^References^** | **Original research** | | | **INRA (13)** | | | | | | | | | | | | | | **NorFor (14)** | | | | | | |
| --- | --- | --- | --- | --- | --- | --- | --- | --- | --- | --- | --- | --- | --- | --- | --- | --- | --- | --- | --- | --- | --- | --- | --- | --- |
|  | **SBL** | **SBPS** | **SBPW** | **Barley^a^** | **Maize^a^** | **Oats^a^** | **Rye^a^** | **Triticale^a^** | **Wheat durum^a^** | **Wheat soft^a^** | **Rapeseed Meal, oil <5 %^a^** | **Soybean meal, oil <5, 50 % protein + oil ^a^** | **Peas^a^** | **sunflower meal, oil <5 %, dehulled^a^** | **faba beans, coloured flowers^a^** | **linseed whole^a^** | **linseed meal, oil < 5% ^a^** | **white clover^b,c^** | **red clover^b,d^** | **silage grass clover^b, e^** | **clover grass hay, high OMD ^b,f^** | **straw, spring barley^b,g^** | **Italian ryegrass, spring, early^b,h^** | **Grass, mixed meadow. Medium dig.^b,1^** |
| **His** | 3.4 | 5.6 | 2.5 | 2.6 | 2.6 | 2.3 | 2.1 | 2.7 | 3.7 | 2.9 | 10.5 | 14.7 | 5.9 | 7.4 | 7.5 | 5.2 | 9.7 | 5.4 | 5.9 | 2.4 | 1.4 | 0.7 | 5.2 | 2.4 |
| **Arg** | 6.0 | 31.4 | 4.1 | 5.5 | 4.2 | 7.2 | 5.0 | 6.1 | 7.9 | 6.3 | 22.1 | 40.3 | 19.9 | 24.6 | 27.9 | 22.6 | 34.1 | 12.2 | 12.0 | 4.1 | 4.1 | 1.9 | 12.1 | 3.8 |
| **Ser** | 8.9 | 15.9 | 4.4 | 4.7 | 4.4 | 5.3 | 4.4 | 5.2 | 8.2 | 6.3 | 16.7 | 25.4 | 10.9 | 12.8 | 14.9 | 11.4 | 17.2 | 12.6 | 11.6 | 5.5 | 4.8 | 1.9 | 10.2 | 5.1 |
| **Asp** | 19.8 | 34.6 | 20.6 | 6.7 | 5.7 | 9.3 | 7.5 | 7.6 | 8.2 | 6.5 | 26.9 | 62.4 | 27.2 | 26.6 | 35.2 | 22.8 | 35.1 | 31.5 | 24.2 | 11.5 | 8.8 | 4.7 | 24.4 | 11.1 |
| **Glu** | 13.0 | 39.0 | 19.6 | 25.7 | 16.3 | 18.4 | 22.1 | 27.7 | 50.7 | 36.3 | 64.8 | 98.7 | 38.1 | 57.4 | 51.9 | 48.7 | 72.9 | 27.8 | 30.1 | 10.4 | 10.7 | 4.7 | 23.9 | 10.9 |
| **Thr** | 7.2 | 8.5 | 4.3 | 3.9 | 3.3 | 3.8 | 3.4 | 3.8 | 4.8 | 3.8 | 16.4 | 20.9 | 8.9 | 11.2 | 10.5 | 10.0 | 13.9 | 11.7 | 10.6 | 5.2 | 4.5 | 1.9 | 10.6 | 4.8 |
| **Gly** | 7.7 | 12.0 | 4.6 | 4.6 | 3.4 | 5.5 | 4.4 | 5.0 | 6.5 | 5.2 | 16.8 | 23.1 | 10.3 | 17.4 | 12.7 | 13.5 | 21.6 | 13.4 | 13.1 | 6.4 | 5.1 | 2.2 | 12.3 | 5.7 |
| **Ala** | 7.4 | 10.7 | 4.5 | 4.7 | 6.5 | 5.1 | 4.3 | 4.9 | 5.6 | 4.6 | 16.5 | 24.0 | 10.3 | 13.2 | 12.2 | 10.5 | 17.1 | 14.1 | 14.5 | 9.0 | 5.8 | 2.7 | 16.0 | n/a |
| **Pro** | 13.7 | 9.2 | 3.6 | 11.9 | 8.2 | 6.6 | 9.8 | 10.1 | 17.3 | 12.6 | 23.0 | 27.4 | 9.7 | 12.8 | 12.2 | 8.3 | 13.5 | 16.3 | 11.3 | 9.4 | 5.2 | 4.4 | 11.6 | 7.1 |
| **Tyr** | 5.8 | 6.9 | 3.8 | 3.2 | 3.7 | 3.8 | 2.5 | 3.5 | 5.2 | 3.9 | 10.8 | 19.4 | 7.3 | 7.2 | 9.4 | 6.3 | 8.8 | 8.9 | 7.6 | 3.5 | 2.7 | 1.4 | 8.1 | 4.2 |
| **Met** | 1.7 | 1.4 | 0.9 | 1.9 | 1.9 | 1.9 | 1.6 | 2.0 | 2.6 | 2.0 | 7.7 | 7.9 | 2.3 | 6.9 | 2.1 | 4.4 | 6.8 | 2.8 | 3.3 | 2.1 | 1.8 | 0.7 | 4.6 | 1.8 |
| **Val** | 6.9 | 6.4 | 4.4 | 5.7 | 4.4 | 5.7 | 4.8 | 5.5 | 7.1 | 5.6 | 19.5 | 26.6 | 11.0 | 14.9 | 13.3 | 11.8 | 18.5 | 13.8 | 12.8 | 7.9 | 5.0 | 2.5 | 13.0 | 7.1 |
| **Trp** | 1.1 | 2.3 | 0.8 | 1.4 | 0.6 | 1.4 | 0.9 | 1.4 | 1.8 | 1.5 | 4.7 | 7.7 | 2.0 | 3.9 | 2.4 | 4.0 | 5.5 | 0.0 | 2.6 | 2.0 | 0.3 | 0.6 | n/a | 1.6 |
| **Cys** | 1.7 | 2.3 | 1.5 | 2.6 | 2.2 | 3.6 | 2.3 | 3.0 | 3.6 | 3.1 | 9.1 | 8.7 | 3.2 | 5.2 | 3.7 | 4.4 | 6.7 | 2.7 | 2.3 | 1.2 | 1.7 | 0.5 | 3.3 | 0.9 |
| **Phe** | 7.6 | 8.8 | 3.3 | 5.5 | 4.3 | 5.4 | 4.3 | 5.0 | 8.0 | 5.9 | 14.7 | 27.9 | 11.0 | 13.5 | 12.4 | 12.0 | 17.6 | 12.9 | 10.9 | 5.9 | 4.3 | 2.0 | 12.1 | 5.9 |
| **Ile** | 5.1 | 5.4 | 1.5 | 4.1 | 3.3 | 4.1 | 3.4 | 4.4 | 5.9 | 4.5 | 15.3 | 25.1 | 9.8 | 12.5 | 12.0 | 10.7 | 15.5 | 10.5 | 9.5 | 6.0 | 3.7 | 1.9 | 10.3 | 5.5 |
| **Leu** | 11.9 | 16.8 | 5.9 | 7.7 | 10.8 | 7.9 | 6.0 | 7.3 | 11.1 | 8.5 | 26.0 | 42.2 | 166.0 | 18.7 | 22.4 | 14.3 | 21.7 | 19.8 | 12.5 | 9.6 | 6.4 | 3.5 | 18.2 | 8.9 |
| **Lys** | 10.2 | 11.3 | 4.8 | 4.3 | 2.7 | 4.5 | 3.9 | 4.5 | 4.3 | 3.6 | 20.4 | 34.3 | 17.0 | 11.2 | 8.8 | 8.8 | 14.3 | 12.5 | 13.3 | 5.5 | 4.1 | 1.8 | 11.9 | 5.1 |

**^a^** Values have been taken from INRA feed tables

^b^ Values have been taken from NorFor feed tables, initially presented as g/100 of crude protein they were converted to g/kg DM

^c^ White clover from the NorFor tables (code 006-0093) was used

^d^ Red clover from the NorFor tables (code 006-0093) was used

^e^ Silage grass clover from the NorFor tables (code 006-0165) was used

^f^ Clover grass hay, with high Organic Mater Digestbility (OMD) from the NorFor tables (code 006-0403) was used

^g^ Straw, spring barley from the NorFor tables (code 006-0386) was used

^h^ Italian ryegrass, spring, early from the NorFor tables (code 006-0084) was used

^i^ Grass, mixed meadow with medium digestibility (dig) from the NorFor tables (code 006-0503) was used

**Table S11.** Fatty acid concentration of sea buckthorn leaves (SBL), sea bucktorn pomace with seeds (SBPS) and sea buckthorn pomace without seeds (SBPW) and conventional feed ingredients, expressed as g/kg DM

| **References** | **Original research** | | | | | **INRA (13)** | | | | | | | | | | | | | **NorFor (14)** | | | | | | |
| --- | --- | --- | --- | --- | --- | --- | --- | --- | --- | --- | --- | --- | --- | --- | --- | --- | --- | --- | --- | --- | --- | --- | --- | --- | --- |
|  | **SBL** | **SBPS** | **SBPW** | **Barley^a^** | **Maize^a^** | | **Oats^a^** | **Rye^a^** | **Triticale^a^** | **Wheat durum^a^** | **Wheat soft^a^** | **Rapeseed Meal, oil <5 %^a^** | **Soybean meal, oil <5, 50 % protein + oil ^a^** | **Peas^a^** | **sunflower meal, oil <5 %, dehulled^a^** | **faba beans, coloured flowers^a^** | **linseed whole^a^** | **linseed meal, oil < 5% ^a^** | **white clover^b,c^** | **red clover^b,d^** | **silage grass clover^b, e^** | **clover grass hay, high OMD ^b,f^** | **straw, spring barley^b,g^** | **Italian ryegrass, spring, early^b,h^** | **Grass, mixed meadow. Medium dig.^b,1^** |
| **C16:0** | 0.57 | 2.08 | 5.82 | 3.10 | 3.80 | | 8.00 | 1.60 | 1.80 | 2.70 | 2.10 | 0.90 | 1.40 | 1.60 | 1.00 | 2.10 | 20.10 | 2.00 | 2.97 | 2.89 | 1.88 | 2.37 | 0.55 | 6.93 | 2.94 |
| **C16:1c9** | 0.17 | 1.75 | 6.00 | 0.03 | 0.00 | | 0.10 | 0.08 | 0.09 | 0.06 | 0.05 | 0.04 | 0.01 | 0.02 | 0.03 | 0.01 | 0.30 | 0.01 | n/a | 0.00 | 0.00 | n/a | 0.00 | 0.00 | 0.00 |
| **c18:1c9** | 0.15 | 0.89 | 0.85 | 1.90 | 9.30 | | 18.70 | 1.90 | 1.40 | 2.30 | 1.80 | 11.70 | 3.00 | 2.80 | 3.20 | 3.10 | 72.00 | 3.10 | 0.86 | 1.23 | 0.32 | n/a | 0.08 | 0.93 | 0.51 |
| **C18:1c11** | 0.06 | 0.44 | 1.04 | n/a | n/a | | n/a | n/a | n/a | n/a | n/a | n/a | n/a | n/a | n/a | n/a | n/a | n/a | n/a | 0.00 | 0.00 | n/a | 0.00 | 0.00 | 0.00 |
| **c18:2n6** | 0.17 | 2.46 | 2.48 | 8.00 | 21.20 | | 19.90 | 6.10 | 6.40 | 8.60 | 6.70 | 3.90 | 6.90 | 5.40 | 10.20 | 6.10 | 52.50 | 6.10 | 2.76 | 3.56 | 1.92 | 2.04 | 0.76 | 4.80 | 3.02 |
| **c18:3n3** | 0.20 | 1.39 | 0.31 | 1.30 | 0.50 | | 0.80 | 0.70 | 0.70 | 0.90 | 0.70 | 1.80 | 0.90 | 1.00 | 0.05 | 0.40 | 192.00 | 0.40 | 9.57 | 6.59 | 6.21 | 6.08 | 1.62 | 19.52 | 9.73 |
| **c22:0** | 0.11 | 0.02 | 0.04 | 0.00 | 0.00 | | 0.03 | 0.00 | 0.00 | 0.00 | 0.00 | 0.00 | 0.06 | 0.03 | 0.05 | 0.05 | 0.40 | 0.05 | n/a | 0.00 | 0.00 | n/a | 0.00 | 0.00 | 0.00 |

**^a^** Values have been taken from INRA feed tables

^b^ Values have been taken from NorFor feed tables, initially presented as g/100 of crude fat they were converted to g/kg DM

^c^ White clover from the NorFor tables (code 006-0093) was used

^d^ Red clover from the NorFor tables (code 006-0093) was used

^e^ Silage grass clover from the NorFor tables (code 006-0165) was used

^f^ Clover grass hay, with high Organic Mater Digestbility (OMD) from the NorFor tables (code 006-0403) was used

^g^ Straw, spring barley from the NorFor tables (code 006-0386) was used

^h^ Italian ryegrass, spring, early from the NorFor tables (code 006-0084) was used

^i^ Grass, mixed meadow with medium digestibility (dig) from the NorFor tables (code 006-0503) was used

**Table S12.** Concentration of vitamins in sea buckthorn leaves (SBL), sea bucktorn pomace with seeds (SBPS) and sea buckthorn pomace without seeds (SBPW)and conventional feed ingredients expressed as mg/kg DM

| References | **Original research** | | | **INRA (13)** | | | | | | | | | | | | | | **NorFor (14)** | | | | | | |
| --- | --- | --- | --- | --- | --- | --- | --- | --- | --- | --- | --- | --- | --- | --- | --- | --- | --- | --- | --- | --- | --- | --- | --- | --- |
|  | **SBL** | **SBPS** | **SBPW** | **Barley^a^** | **Maize^a^** | **Oats^a^** | **Rye^a^** | **Triticale^a^** | **Wheat durum^a^** | **Wheat soft^a^** | **Rapeseed Meal, oil <5 %^a^** | **Soybean meal, oil <5, 50 % protein + oil ^a^** | **Peas^a^** | **sunflower meal, oil <5 %, dehulled^a^** | **faba beans, coloured flowers^a^** | **linseed whole^a^** | **linseed meal, oil < 5% ^a^** | **white clover^b,c^** | **red clover^b,d^** | **silage grass clover^b, e^** | **clover grass hay, high OMD ^b,f^** | **straw, spring barley^b,g^** | **Italian ryegrass, spring, early^b,h^** | **Grass, mixed meadow. Medium dig.^b,1^** |
| **Vit A^i^** | <0.02 | <0.05 | <0.02 | 0.0008 | 0.0008 | 0 | 0 | 0 | 0 | 0 | n/a | n/a | n/a | n/a | n/a | 0 | n/a | n/a | n/a | 0.004 | n/a | n/a | 0.03 | 0.02 |
| **β carotene** | 11.5 | 151.3 | 257.9 | n/a | n/a | n/a | n/a | n/a | n/a | n/a | n/a | n/a | n/a | n/a | n/a | n/a | n/a | n/a | n/a | 30 | 60.0 | n/a | 250.0 | 150.0 |
| **Vit D^i^** | <0.01 | <0.005 | <0.01 | 0 | 0 | 0 | 0 | 0 | 0 | 0 | 0 | 0 | 0 | 0 | 0 | 0 | 0 | 0 | 0 | 0 | 0.0 | 0.0 | 0.0 | 0.0 |
| **Vit E** | 335.4 | 380.0 | 720.5 | 18.7 | 19.8 | 13.6 | 11.5 | 1.1 | n/a | 17.5 | 15.4 | 4.6 | 4.8 | 13.3 | 0.6 | 3.4 | 15 | n/a | n/a | n/a | 13.4 | n/a | 100.7 | 53.7 |
| **Vit B1** | <0.1 | 9.08 | <0.1 | 5.1 | 4.1 | 6.6 | 3.9 | 3.6 | n/a | 5.1 | 3.9 | 3.1 | 5.2 | 37.7 | 6.5 | 13.3 | n/a | n/a | n/a | n/a | n/a | n/a | n/a | n/a |
| **Vit B2** | 3.1 | 11.2 | 0.4 | 1.8 | 1.6 | 1.8 | 2.4 | 1.8 | n/a | 1.3 | 4.2 | 3.3 | 2.1 | 3.6 | 3.8 | 2.2 | n/a | n/a | n/a | n/a | n/a | n/a | n/a | n/a |
| **Vit B3** | 32.5 | 39.7 | 4.3 | 60.9 | 24.0 | 19.2 | 34.5 | 21.0 | n/a | 61.9 | 187.0 | 24.7 | 28.6 | 222.0 | 32.9 | 33.5 | n/a | n/a | n/a | n/a | n/a | n/a | n/a | n/a |
| **Vit B5** | 0.0 | 14.2 | 14.3 | 8.6 | 6.7 | 9.5 | 16.1 | 6.7 | n/a | 12.2 | 10.3 | 16.2 | 16.4 | 11.1 | 11.4 | 108.0 | n/a | n/a | n/a | n/a | n/a | n/a | n/a | n/a |
| **Vit B6** | 22.9 | 13.1 | 7.4 | 4.6 | 5.3 | 5.2 | 3.3 | 15.9 | n/a | 3.7 | 11.9 | 7.3 | 4.7 | 14.4 | 4.3 | 6.8 | n/a | n/a | n/a | n/a | n/a | n/a | n/a | n/a |

**^a^** Values have been taken from INRA feed tables

^b^ Values have been taken from NorFor feed tables

^c^ White clover from the NorFor tables (code 006-0093) was used

^d^ Red clover from the NorFor tables (code 006-0093) was used

^e^ Silage grass clover from the NorFor tables (code 006-0165) was used

^f^ Clover grass hay, with high Organic Mater Digestbility (OMD) from the NorFor tables (code 006-0403) was used

^g^ Straw, spring barley from the NorFor tables (code 006-0386) was used

^h^ Italian ryegrass, spring, early from the NorFor tables (code 006-0084) was used

^i^ Grass, mixed meadow with medium digestibility (dig) from the NorFor tables (code 006-0503) was used

References

1. Christie WW. *Gas chromatography and lipids: a practical guide.* 1st ed. Oxford: Oily Press; 1989.
2. Kuoppala K, Vattulainen J, Perttilä S, Saastamoinen M, Rinne M. (2024). Finnish feed tables and nutrient requirements: ruminants, pigs, poultry, horses. A report on Natural Resources and Bioeconomy Studies 70/2024. Helsinki, Finland: Natural Resources Institute Finland..
3. National Research Council (NRC). (2007). Nutrient requirements of horses. 6th ed. Washington, DC: National Academies Press.
4. Tikk H and Piirsalu M. (1997). Põllumajanduslindudele soovitatavad söötmisnormid Eestis. Tartu, Estonia: Eesti Linnukasvatajate Selts
5. McDonald P, Edwards RA, Greenhalgh JFD, Morgan CA, Sinclair LA, and Wilkinson RG. (2011). Animal nutrition. Essex, UK: Pearson Education Limited.
6. Tybirk P, Sloth NM, Kjeldsen NIELS, Weber N. (2020). Danish nutrient standards. SEGE S Danish Pig Research Centre.
7. National Research Council (NRC), Division on Earth, and Committee on Nutrient Requirements of Swine. (2012). Nutrient requirements of swine. Washington, DC: National Academies Press.
8. Oll Ü, Tölp S, et al. (1995). Põllumajandusloomade söötmisnormid koos söötade tabelitega [Feeding standards of farm animals with feed tables]. Tartu, Estonia: Eesti Põllumajandusülikool.
9. Volden H, editor. (2011). NorFor—The Nordic feed evaluation system. Wageningen, The Netherlands: Wageningen Academic Publishers.
10. National Research Council (NRC). (2021). Nutrient requirements of dairy cattle. 8th ed. Washington, DC, USA: National Academies Press.
11. Penn State Extension (2023). Total Mixed Rations for Dairy Cows. Available online at: <https://extension.psu.edu/total-mixed-rations-for-dairy-cows> [accessed on May 18, 2025]
12. Eesti Maaülikool. Veterinaarmeditsiini ja loomakasvatuse instituudi (n.d.) Söötmisteaduse õppetooli projektid. Uuendnoorkarja söötmisstrateegia loomine lüpsikarja efektiivseks ja jätkusuutlikuks taastootmiseks**.** Available online at: <https://vl.emu.ee/sootmisteaduse-oppetooli-projektid> [accessed on: May 15, 2025]
13. INRA (n.d). The INRAE-CIRAD-AFZ tables. Available online at: <https://www.feedtables.com/content/tables> [Accessed June 21, 2025].
14. NorFor (n.d.). Feed tables. Available online at: <https://feedstuffs.norfor.info/> [Accessed June 21, 2025].
